# Navigating uncertainty in environmental DNA detection of a nuisance marine macroalga

**DOI:** 10.1371/journal.pone.0318414

**Published:** 2025-02-04

**Authors:** Patrick K. Nichols, Kaʻuaʻoa M. S. Fraiola, Alison R. Sherwood, Brian B. Hauk, Keolohilani H. Lopes, Colt A. Davis, James T. Fumo, Chelsie W. W. Counsell, Taylor M. Williams, Heather L. Spalding, Peter B. Marko

**Affiliations:** 1 School of Life Sciences, University of Hawaiʻi at Mānoa, Honolulu, HI, United States of America; 2 United States Geological Survey, Honolulu, HI, United States of America; 3 National Oceanic and Atmospheric Administration, Honolulu, HI, United States of America; 4 Natural Resources and Environmental Management, College of Tropical Agriculture and Human Resources, University of Hawaiʻi at Mānoa, Honolulu, HI, United States of America; 5 Cooperative Institute for Marine and Atmospheric Research, University of Hawaiʻi at Mānoa, Honolulu, HI, United States of America; 6 Department of Biology, University of Alabama at Birmingham, Birmingham, AL, United States of America; 7 Department of Biology, College of Charleston, Charleston, SC, United States of America; Jinan University, CHINA

## Abstract

Early detection of nuisance species is crucial for managing threatened ecosystems and preventing widespread establishment. Environmental DNA (eDNA) data can increase the sensitivity of biomonitoring programs, often at minimal cost and effort. However, eDNA analyses are prone to errors that can complicate their use in management frameworks. To address this, eDNA studies must consider imperfect detections and estimate error rates. Detecting nuisance species at low abundances with minimal uncertainty is vital for successful containment and eradication. We developed a novel eDNA assay to detect a nuisance marine macroalga across its colonization front using surface seawater samples from Papahānaumokuākea Marine National Monument (PMNM), one of the world’s largest marine reserves. *Chondria tumulosa* is a cryptogenic red alga with invasive traits, forming dense mats that overgrow coral reefs and smother native flora and fauna in PMNM. We verified the eDNA assay using site-occupancy detection modeling from quantitative polymerase chain reaction (qPCR) data, calibrated with visual estimates of benthic cover of *C*. *tumulosa* that ranged from < 1% to 95%. Results were subsequently validated with high-throughput sequencing of amplified eDNA and negative control samples. Overall, the probability of detecting *C*. *tumulosa* at occupied sites was at least 92% when multiple qPCR replicates were positive. False-positive rates were 3% or less and false-negative errors were 11% or less. The assay proved effective for routine monitoring at shallow sites (less than 10 m), even when *C*. *tumulosa* abundance was below 1%. Successful implementation of eDNA tools in conservation decision-making requires balancing uncertainties in both visual and molecular detection methods. Our results and modeling demonstrated the assay’s high sensitivity to *C*. *tumulosa*, and we outline steps to infer ecological presence-absence from molecular data. This reliable, cost-effective tool enhances the detection of low-abundance species, and supports timely management interventions.

## Introduction

Nuisance species harm biodiversity through a variety of ecological mechanisms, such as landscape homogenization, which can in turn lead to loss of ecosystem resilience and functionality [1]. Managing nuisance species often incurs substantial resources for prevention, eradication, and restoration [2, 3], with high economic and social costs. These impacts include loss of recreational activity and damage to culturally-important species and ecosystems [4, 5]. The cost and effort of detecting nuisance species is inversely proportional to their abundance [6], making detecting nascent introductions along a colonization front a unique challenge for resource managers.

Early detection of nuisance species is crucial for containment [7], but requires reliable, large-scale, and cost-effective monitoring, likely dependent on new technology [8, 9]. Environmental DNA (eDNA) can enhance biomonitoring of nuisance species by isolating and analyzing genetic material shed by an organism into their environment [10, 11]. Collected eDNA is then amplified and measured using either metabarcoding of bulk samples with universal primers or qPCR with species-specific primers. qPCR has successfully identified numerous aquatic nuisance species from eDNA [11–16]. Additionally, eDNA assessments can be orders of magnitude more sensitive than traditional observation methods [10, 17, 18]. By enabling precise, efficient, and reliable eDNA detection of low-abundance organisms with high taxonomic certainty, eDNA fundamentally shifts both how and by whom ecosystems are monitored [19].

Although eDNA methods are commonly portrayed as straightforward to implement, they require expertise in data analysis and interpretation. The rapidly expanding field of eDNA analysis can be difficult to navigate for non-specialists, especially given the lack of standardized procedures for sample handling, replication, negative controls, workflows, and the validation of positives [20–25]. Uncertainty in molecular detection methods can be oversimplified, overlooked [26], or poorly communicated by specialists [27, 28], particularly in early detection of rare organisms, which is more error-prone as compared to widely-established populations [29, 30]. Without project-specific experimental design and analysis, results can be misinterpreted, hindering the production of clear, actionable evidence for management decisions [28, 31, 32].

Interpretation of imperfect detections requires consideration of each source of error inherent in the survey method. Traditional biodiversity monitoring methods that involve direct observations of target organisms also face detection biases [33–35]. Uncertainty in eDNA biomonitoring studies arises from a nested hierarchy of potential latent process errors (e.g., unavoidable errors intrinsic to an experimental design) that start with the collection of multiple samples in the field and end with analysis of multiple replicates in a laboratory [36–38]. For example, target eDNA may be present at a given site, yet fail to be collected in a sample due to large filter pore size [39, 40] or amplified in a PCR replicate due to inhibition [41]. In response, statistical modeling of presence-absence data using imperfect detections has been extensively developed for direct observations of species [42–46]. It is therefore critical that eDNA studies similarly account for imperfect detections and estimate associated error rates.

eDNA-based occupancy detection models have been extended to provide a statistical framework for estimating eDNA detection probabilities and associated errors at the site, sample, and replicate levels [37, 47, 48]. Both traditional site occupancy and eDNA-based occupancy models infer true occupancy while accounting for imperfect detection data. However, models using eDNA detections must also distinguish ecological presence (i.e., target species present at a site) and detection presence (i.e., eDNA is detected, but the target species may not be present locally). Augmentation of the models with unambiguous detection data from other sources (i.e., those that don’t produce false-positives, although false-negatives may still occur) can confirm species presence to distinguish true detections from false ones [38, 49]. These analyses assume the true occupancy state of a site (presence or absence) is unlikely to change throughout repeated surveys of several sites [50]. eDNA datasets typically employ near-simultaneous collections of multiple environmental samples from a site and are therefore suitable for occupancy analyses [36]. Such modeling improves inferences about species distributions [15, 51, 52] by estimating the probability of occupancy (ѱ), eDNA capture (θ) in field samples, and detection (p) in laboratory PCR replicates [37, 38]. Identifying the error associated with the molecular detections of nuisance species is vital for demonstrating the accuracy of eDNA techniques for early detection and communicating uncertainty to stakeholders [31].

The nested sampling inherent in eDNA surveys (replicates amplified from samples collected from multiple sites) creates a complex hierarchy of error sources often confused by end users, but which are vital for understanding detection uncertainty [30]. For instance, the term “false-positive” can refer to site-specific level errors (i.e., eDNA detected at a site where the target species is presumed absent) or laboratory-level errors (i.e., a positive PCR technical replicate when eDNA is absent in a sample). Although both are “false positives”, we follow Darling et al. [30] in the use of terminology to distinguish among different types of false-positives and negatives within the hierarchy of eDNA sampling (**Box 1**). First, “presumed-positive” or “presumed-negative” are sites for which presence-absence is inferred with eDNA without corroboration from non-eDNA observations. Second, a “false-positive inference” is a presumed-positive site for which non-eDNA methods estimate species absence. Third, a “false-positive test” is a sample-level error, such as contamination or PCR errors. Lastly, a “false-negative test” is when non-eDNA presence is inferred despite no eDNA detection. We explicitly model the probability of errors at each hierarchical step of our eDNA analysis to clarify uncertainty for molecular detection of nuisance species.

**Box 1. Terminology used throughout for distinguishing errors in the hierarchical framework of environmental DNA sampling, following Darling et al. (2021)**

**Table:** 

**Presumed-positive:**	eDNA presence is presumed in the absence of non-eDNA observations
**Presumed-negative**	eDNA absence is presumed in the absence of non-eDNA observations
**False-positive inference**	eDNA presence is presumed although non-eDNA methods estimate species absence with reasonable confidence
**False-positive test**	eDNA presence is due to sample-level error, such as contamination or PCR errors
**False-negative test**	no eDNA detection and non-eDNA observations confirm presence

**Fig 1:**
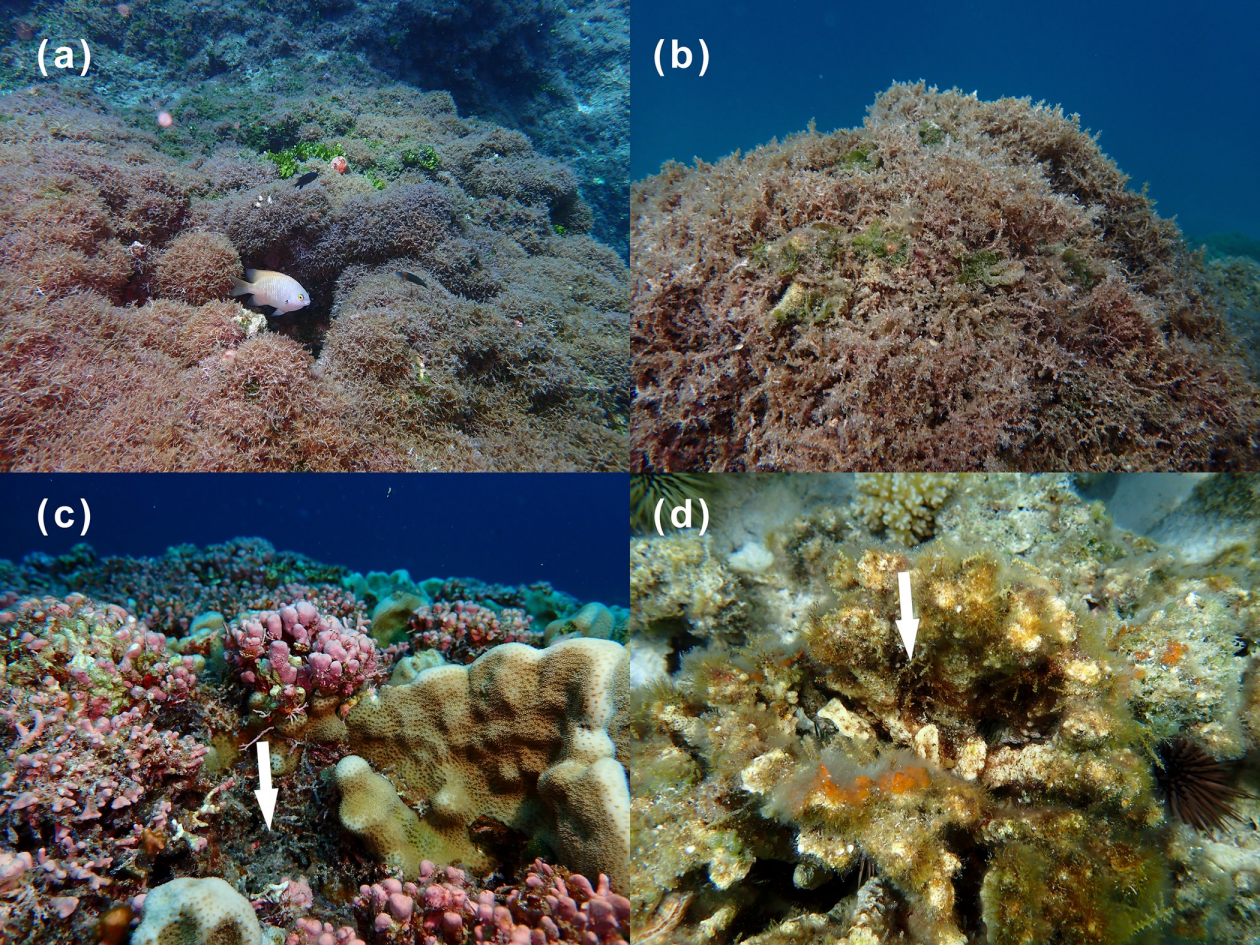
Visual observations of benthic cover. *Chondria tumulosa* benthic cover varied widely, from 95% in areas where it formed extensive mats (Manawai, a-b) to 5% or less in locations where it grew cryptically, concealed within reef interstices or among native taxa (Manawai, c; Kuaihelani, d). Photo credits: H.L. Spalding.

Detecting, assessing, and mitigating nuisance species in remote and ecologically-sensitive locations is costly. Papahānaumokuākea Marine National Monument (PMNM), a marine conservation area and UNESCO world heritage site, is threatened by the cryptogenic red alga *Chondria tumulosa* (Rhodomelaceae, Rhodophyta). First observed in PMNM at Manawai (also known as Pearl and Hermes Atoll) in 2015–2016 forming expansive mats [53, 54], *C*. *tumulosa* was later discovered at neighboring Kuaihelani (Midway Island) and Hōlanikū (Kure Atoll) in low-abundance algal-turf matrices, making visual detection challenging (**Fig 1**). Remote islands are particularly vulnerable to nuisance species introductions [55, 56] and the large size and remoteness of PMNM’s reefs have made early visual detection of *C*. *tumulosa* difficult. Although visual surveys are ideal to establish the presence of *C*. *tumulosa*, they are costly, require taxonomic expertise, and may miss cryptic individuals or life stages [12, 57, 58]. In an effort to minimize the costs associated with biomonitoring, improve detection sensitivity, and identify early colonization events, eDNA has been gaining traction as a tool for biosecurity management [9, 12, 59].

The expanding spatial distribution of *C*. *tumulosa* in PMNM is an ideal system for testing aquatic molecular detection tools. Here, we document the development of a qPCR assay for *C*. *tumulosa* and used occupancy modeling with eDNA and visual survey data to enhance detections and establish objective error rates for implementing the surface water eDNA assay across varying site depths. However, modeling alone could not definitively determine presence-absence of *C*. *tumulosa* at all sites. Visualization of raw qPCR data, negative controls, and validation with high-throughput sequencing resolved uncertainties and demonstrates the difficulty of devising eDNA sampling strategies for low-abundance nuisance species.

## Methods

### Ethics statement

Field work was conducted under PMNM permits (PNMN-2019-001, PMNM-2021-016, PMNM-2021-019, PMNM-2022-011, and PMNM-2023-01).

### Sampling

Sixty-three sites across the Hawaiian Archipelago were sampled for *C*. *tumulosa* eDNA between 2021–2023 (**S1 Table**). 2-L replicate seawater samples (n = 160, representing 2–3 replicates per site) were collected from the top meter of the water column at all sites. Each 2-L biological sample and a control (1-L tap water or DI water) were shaken, filtered through mixed cellulose ester filters (Millipore; diameter: 47 mm; pore size: 0.22 μm) on a peristaltic pump (Cole-Parmer, USA), and frozen in liquid nitrogen. DNA was extracted (DNeasy Blood & Tissue kit, Qiagen, USA) from thawed filters as previously described [60].

Water sample filtration was performed shipboard during expeditions to PMNM while DNA extraction and amplification were conducted in physically isolated laboratory spaces at the University of Hawaiʻi at Mānoa. All laboratory surfaces and equipment were sterilized using a 10% bleach solution before and after processing samples. Water collection containers were sterilized with 10% bleach for 12 h, air dried, and rinsed with surface seawater at each new location prior to sampling. Equipment contamination was monitored using bleach-sterilized filtration equipment and a 1-L tap water or DI water equipment blank (EB) filtered between samples from new sites. PCR contamination was monitored with triplicate PCR negatives (no-template controls, NTC).

**Fig 2:**
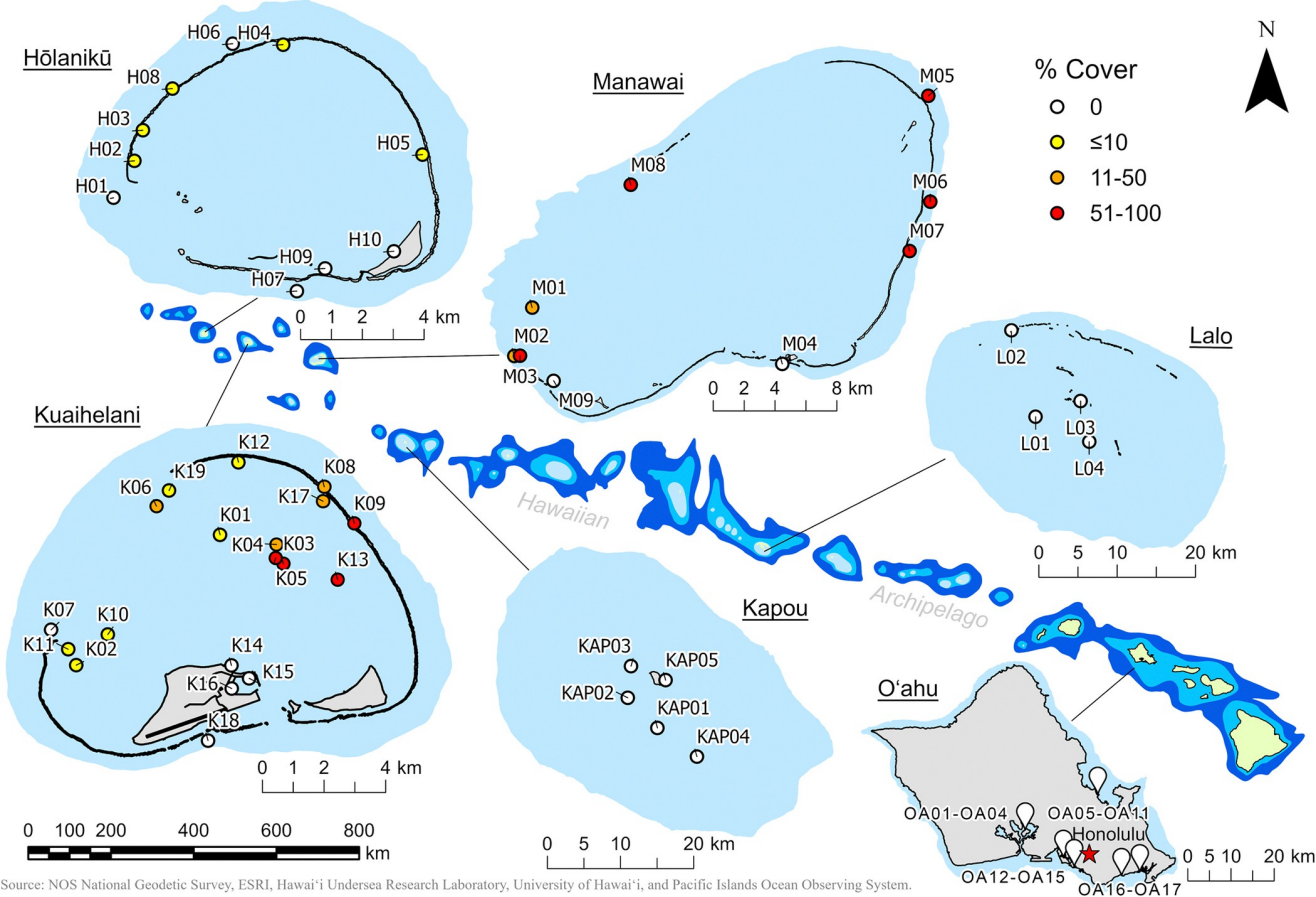
Map of sampling locations throughout the Hawaiian Archipelago. Seawater environmental DNA (eDNA) samples were collected at 63 locations throughout the Hawaiian Archipelago. *Chondria tumulosa* presence or absence in Papahānaumokuākea Marine National Monument was confirmed by visual surveys at Hōlanikū (Kure Atoll), Kuaihelani (Midway Island), Manawai (Pearl & Hermes Atoll), Kapou (Lisianski Island), and Lalo (French Frigate Shoals). Circles on the map are colored according to *C*. *tumulosa* benthic percent cover from visual surveys (white = 0%, yellow = ≤10%, orange = 11–50%, red = 51–100%). White pins on the map represent sites where only eDNA was collected (Oʻahu, “eDNA only”).

Environmental DNA-based occupancy models that include complementary visual data are more robust [37, 48]. Therefore, visual surveys were also conducted via SCUBA diving or skin diving (depending on site depth) immediately following eDNA collection from atolls within PMNM where the species is currently expanding its range (**Fig 2**). Experts in the morphometric identification of *C*. *tumulosa* estimated benthic cover within a 10 m radius (~314 m^2^) at each site, directly below the location of eDNA collection. In this region, where *C*. *tumulosa* is patchily distributed, combining visual data with small-scale variation in target species cover and environmental covariates has the greatest potential to inform the model (see *Site-occupancy detection modeling*, below).

### qPCR assay development and validation

Sixteen species-specific ribulose-1,5-bisphosphate carboxylase/oxygenase large subunit (*rbc*L) primer pairs were developed using alignments of *C*. *tumulosa* and related taxa (Sherwood et al. 2020) and evaluated *in silico* with Primer3 (v2.3.7 using the recommended SantaLucia 1998 formula) in Geneious Prime v2022.1.1 (Biomatters Ltd.) and the National Center for Biotechnology Information (NCBI) primer-BLAST tool (https://www.ncbi.nlm.nih.gov/tools/primer-blast/) using default specificity parameters to ensure species-specific amplification. The four best pairs (T_m_ > 55°C, GC: 40–60%, no secondary structures) matched to five separate *C*. *tumulosa* NCBI entries [53] and one identified as *Yuzurua* sp. (Rhodomelaceae, Rhodophyta: OK209862) from Bermuda [61] (**S2 Table**). Each pair was then tested on *C*. *tumulosa* DNA from tissue samples using a touchdown qPCR amplification profile (**S3 Table**).

Optimal qPCR conditions were determined from triplicate reactions on a CFX96 Touch Real-Time Detection System (Bio-Rad) (**S1 Fig**). The CFX96 calculated critical thresholds of fluorescence (above which detections can be discriminated from background noise), averaged across amplification plates. Assay sensitivity was tested with a standard curve, generated by serially diluting synthetic double-stranded gBlock® DNA fragments (Integrated DNA Technologies, Coralville, IA) (**S4 Table**). With the known concentration of PCR template (in ng μL^-1^) and the length of the synthetic double-stranded DNA fragment (157 bp), the copy numbers were calculated using an online tool recommended by the manufacturer (https://scienceprimer.com/copy-number-calculator-for-realtime-pcr). Triplicate 10-fold serial dilutions ranging from 10^6^ to 10^0^ copies per reaction were amplified using the same cycling parameters as above. The limit of detection (LOD, where 95% of technical replicates amplify) and limit of quantification (LOQ, the lowest initial DNA concentration quantifiable with a coefficient of variation below 35%) was determined using the curve-fitting approach LOD/LOQ script in R v4.3.2 [62, 63]. Percent efficiency was calculated from the standard curve ordinary least squares regression slope on ln-transformed standards, where 100% efficiency corresponded to a doubling of target sequences per cycle [64].

Assay specificity (negatives correctly identified as negative) was tested with serial dilutions (ranging from 10^−1^ to 10^−7^) of 0.57 ± 0.12 ng μL^-1^ close-relative DNA tissue extracts (*Chondria arcuata*, *Chondria* sp., and *Acanthophora spicifera*) [53], which may be found in our survey sites. Only one primer pair amplified *C*. *tumulosa* DNA and not DNA from close relatives, and was used throughout the subsequent analyses (**Table 1**). Each PCR plate contained triplicate serial dilutions of DNA standard, sourced from tissue of the target species as outlined above. Typically, each plate accommodated seven standards (ranging from 10^−1^ to 10^−7^), enabling the generation of a regression line to estimate the quantities of unknown DNA extracts. The efficiency-corrected eDNA starting quantities (SQ) for each sample were calculated using the efficiency, C_q_-value, and fluorescence quantification threshold from each 96-well plate, normalizing unknown sample concentrations with known concentration samples across qPCR runs with different efficiencies [65]. Assay performance was tested *in situ* at sites with visual estimates of *C*. *tumulosa* abundance. Presence-absence was determined two ways: through site-occupancy detection modeling and naïve occupancy assignment by visualizing raw qPCR data amplification and melt curves in R.

**Table 1:** *Chondria tumulosa* rbcL primer sequences.

Name	Length (base pairs)	Sequence (5’->3’)	% GC	T_melt_ (°C)	T_optimal_ (°C)	T_assay_ (°C)
CT_rbcL_F1	20	GCCGTGAATCGTTCTATTGC	50	57.6		
CT_rbcL_R1	24	TCAGCTCTTTCGTACATATTCTCC	41.67	58.2		
Amplified DNA	95	GCCGTGAATCGTTCTATTGCTGCAACTGGAGAAGTAAAAGGTCATTACATGAACGTAACAGCAGCAACTATGGAGAATATGTACGAAAGAGCTGA	42.1	79.7	58.2^a^	54.7

Species-specific primers for the chloroplast ribulose bisphosphate carboxylase large chain (rbcL) gene designed to amplify DNA from *Chondria tumulosa* (GenBank ID: MT039604). ^a^The theoretical optimum annealing temperature (T_optimal_) was calculated using Primer3 (v2.3.7) in Geneious Prime (Biomatters, Ltd.). The actual assay annealing temperature (T_assay_) was determined empirically using a qPCR temperature gradient.

#### Site-occupancy detection modeling

We applied a hierarchical site-occupancy detection model to qPCR detection data [36–38] using the eDNA RShiny application (https://seak.shinyapps.io/eDNA/) for R [66]. This approach integrates eDNA detection data with opportunistic visual survey data while accounting for both false-positive and false-negative errors [38]. In the Bayesian model framework, site-level eDNA detection was inferred using the latent variables for presence and the site occupancy parameter (ψ). The posterior probability of presence reflects the proportion of model iterations where presence of eDNA is inferred at a specific site based on observed data (i.e., eDNA qPCR detection data, visual survey presence-absence data, and habitat covariates), even if it was not visually observed. If the target organism is visually observed, the posterior probability of presence is fixed (presence = 1), but visual absences do not override eDNA-based presence estimates [38].

The occupancy probability represents the likelihood that a new site, with the same covariates, is occupied (i.e., the inherent probability of DNA presence based on site characteristics), providing a broader predictive measure of site suitability. eDNA detections and non-detections are incorporated into the model as observed data that inform the posterior probability of presence for a specific site. These detections also influence the occupancy probability and refine the relationship between covariates and occupancy. We therefore focus on the probability of presence as an indicator of the underlying reality of *C*. *tumulosa* eDNA presence and use the occupancy probability to illustrate the uncertainty associated with imperfect detections across habitat covariates.

Posterior probabilities for *C*. *tumulosa* occupancy at a site (ψ), a sample from an occupied site containing *C*. *tumulosa* eDNA (θ_11_), and a positive qPCR replicate from a sample containing *C*. *tumulosa* eDNA (p_11_) were modeled as a function of habitat covariates in the hierarchical latent process model framework (**S2 Fig**). False-positive inferences at the sample level (θ_10_), false-positive tests at the qPCR replicate level (p_10_), and false-negative tests (1- θ_11_ and 1-p_11_, respectively) were also estimated. One chain, thinned at 20 iterations, was run for 2,000 burn-in iterations, 6,000 additional iterations, using default priors (ψ = 0.5, variance of site occupancy = 4, variance of coefficients = 0.25, significant covariates = 2, θ_11_ = 0.9, θ_10_ = 0.1, p_11_ = 0.9, p_10_ = 0.1). Chain convergence was assessed using the Geweke diagnostic [67] and by visualizing resulting plots using the application (**S3–S8 Figs**). Convergence was reached by increasing the chain length from 2,000 to 6,000 iterations. Visually-estimated presence-absence data were used as direct observations to augment modeling of *C*. *tumulosa* eDNA. We also considered four continuous covariates (*C*. *tumulosa* benthic cover, site depths, and X-Y site coordinates) and one categorical covariate (survey year) for estimation of ψ, θ, and p. Continuous covariates were standardized by subtracting the mean and dividing by the standard deviation for each covariate. Covariate importance was estimated through Bayesian variable selection (presented in [38]) using an Add-Delete-Swap approach and Pólya-Gamma sampling, automated within the eDNA RShiny application. Covariates were considered important predictors if their Posterior Inclusion Probability (PIP) values were greater than 0.5 (i.e., appeared in more than 50% of model iterations).

Performance of qPCR technical replication was evaluated using the posterior conditional probability of species absence given x positive qPCR replicates, 1- ψ(x), and x positive replicates given target eDNA presence, q(x) [38].

### Naïve occupancy

Naïve occupancy from raw qPCR data was indicated at a site when the generalized additive model (GAM) best fit smoother line exceeded the mean critical fluorescence threshold, demonstrating precision across replicates. Sensitivity (sites correctly identified as positive) was determined by comparing congruency between site-level positive detections (sites where GAM smoothers exceeded the fluorescence threshold) and visual estimates from corresponding sites. The probability of inferring naïve occupancy based on qPCR detection thresholds (i.e., the level of agreement among technical qPCR replicates) was modeled using the probability of a true positive qPCR detection (p_11_) from surface eDNA as a function of important habitat covariates (PIP > 0.5). A beta regression was used to model the relationship between p_11_ and important predictors (site depth and benthic cover of *C*. *tumulosa*) using the betareg package in R [68]. The fitted model was then used to expand, predict, and visualize the detection probability contour surface, plotted using metR [69] and ggplot2 in R [70].

### eDNA sequencing

A subset of samples (19 field and 4 control) underwent triplicate qPCR amplifications with Illumina adapters and library preparation at the University of Hawaiʻi at Mānoa (UHM) Microbial Genomics and Analytical Laboratory Core. Pooled equimolar amplicons (including no template controls) were then pair-end sequenced on an Illumina MiSeq platform using the V3 600-cycle chemistry in one flow cell at the Advanced Studies in Genomics and Proteomics facility at UHM. Paired sequence reads (3,040,414) were merged and trimmed in Geneious Prime (Q = 30), followed by dereplication, clustering, and removal of chimeras in VSEARCH [71]. Contaminated reads were removed with microDecon (prop.thresh = 1e-04) in R [72]. Resulting molecular operational taxonomic units (MOTUs) with ≥ 98% sequence identity at the genus level and 100% at the species level were identified with BLASTn searches of the NCBI nucleotide database. Sequence reads were normalized using the eDNA index metric which corrects for differences in sample read depths and amplification efficiencies across sequences [73].

## Results

*Chondria tumulosa* was visually observed at 26 of the 63 sites (**Fig 2**). The highest abundances were on Manawai and Kuaihelani (ranging from 1–95% cover) and lowest abundances were observed at Hōlanikū (only present in < 1% cover). *C*. *tumulosa* was not observed at sites from Kapou and Lalo. Prior surveys of macroalgae have not detected *C*. *tumulosa* around Oʻahu [74]. The optimal annealing temperature was 54.7°C (**S1A Fig**) and all temperatures exhibited single melt peaks (**S1B Fig**). The fitted standard curve generated an R^2^-value of 0.999, slope of -3.79, intercept at 33.6 (predicted C_q_-value with 1 copy of target sequence), and 84% efficiency (**S1C Fig**). The LOD using the curve-fitting approach was 160 copies and the LOQ was 1292 copies. The effective limit of detection when analyzing three qPCR replicates was 118 copies.

### Site-occupancy detection modeling

The hierarchical modeling framework provided estimates of occupancy (ψ), sample capture (θ_11_), qPCR replicate detection (p_11_), and error rates associated with the assay’s use. The baseline (when all covariates equal zero) posterior mean probability of eDNA occupancy was 0.45 (CI: 0.2–0.7) as estimated using the model (**S5 Table**). The mean posterior probability of occupancy for each atoll (i.e., the inherent probability of DNA presence based on all site characteristics within an atoll) was estimated to be 0.79 (CI: 0.52–0.93) for Manawai, 0.71 (CI: 0.50–0.87) for Kuaihelani, 0.55 (CI: 0.29–0.79) for Hōlanikū, 0.31 (CI: 0.12–0.57) for Kapou, 0.13 (CI: 0.03–0.32) for Lalo, and 0.06 (CI: 0–0.19) for Oʻahu.

**Fig 3:**
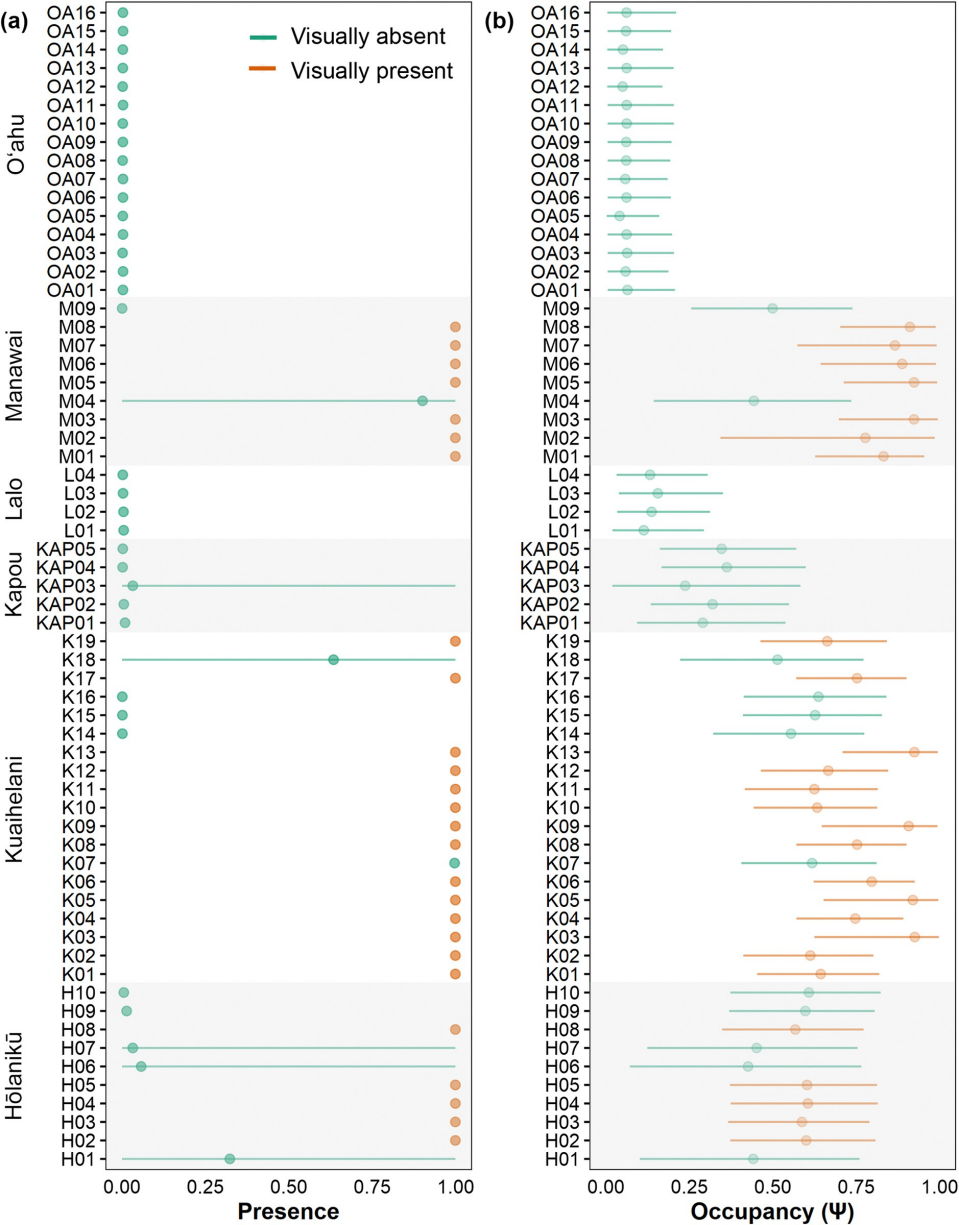
Site-occupancy detection model estimates by site. Posterior mean and 95% credible intervals for (a) the probability of *Chondria tumulosa* presence (probability that a site has *C*. *tumulosa* eDNA based on qPCR detection data, opportunistic visual survey data, and habitat covariates) and (b) the occupancy probability (ψ, the inherent probability of eDNA presence based on site characteristics). Sites are colored by direct visual estimates of *C*. *tumulosa* presence (orange) or absence (green).

**Fig 4:**
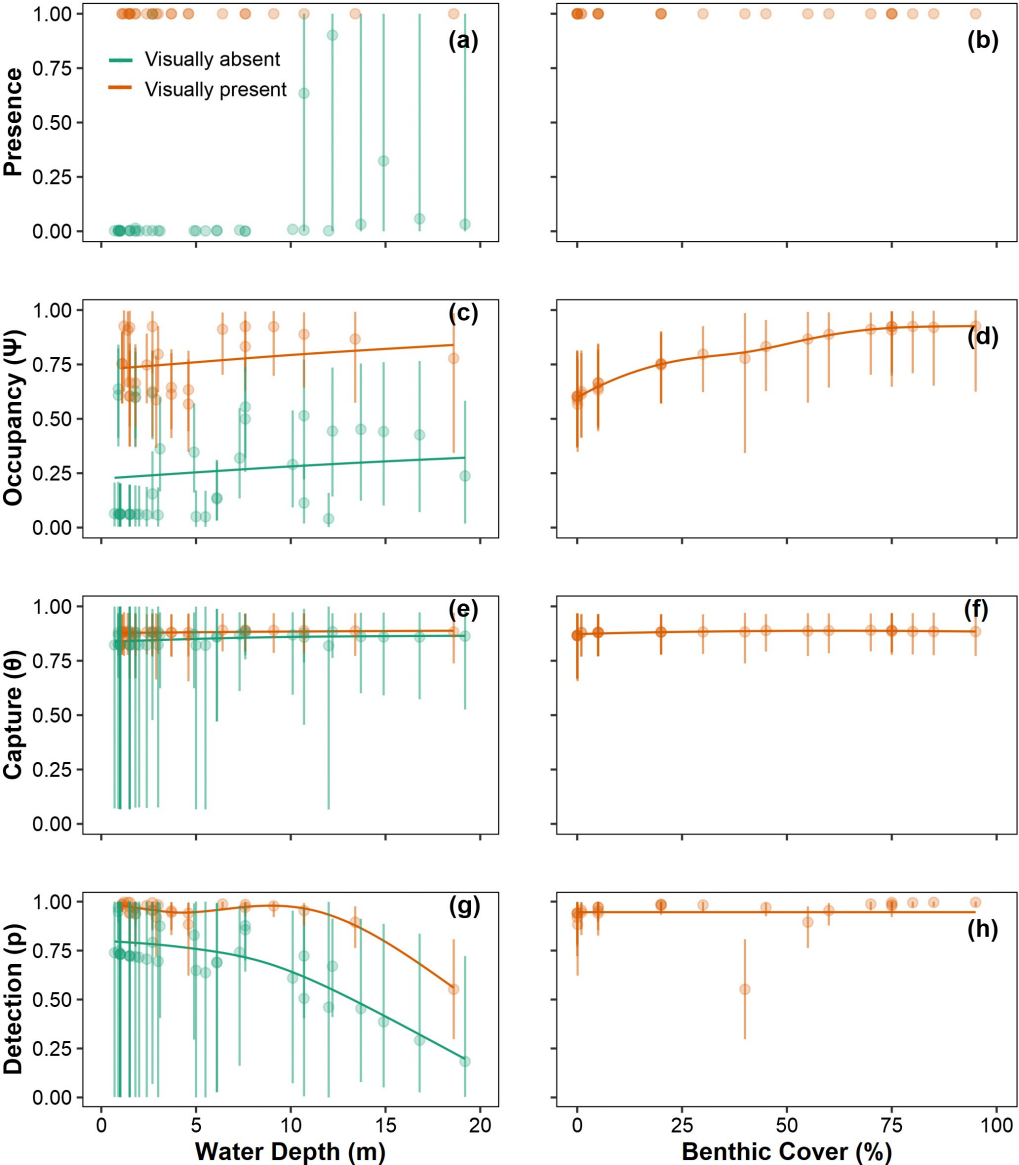
Site-occupancy detection model parameter estimates by site depth and percent benthic cover. Posterior mean and 95% credible intervals for all model parameters: probability of *Chondria tumulosa* presence (probability that a site is occupied based on eDNA qPCR detection data, opportunistic visual survey data, and habitat covariates, panels a-b), occupancy (ψ, inherent probability of eDNA presence based on site characteristics, panels c-d), sample capture given *C*. *tumulosa* eDNA presence at a site (θ_11_, panels e-f), and probability of quantitative polymerase chain reaction (qPCR) replicate detection given *C*. *tumulosa* eDNA presence in a sample (p_11_, panels g-h). Posterior means are colored by direct visual estimates of *C*. *tumulosa* presence (orange) or absence (green). Sites absent of *C*. *tumulosa* are omitted from the right panels (b, d, f, h) for clarity. Trendlines represent generalized additive model smoothers across site depth (range: 1 m– 18 m) and benthic cover (range: < 1%– 95%).

We also estimated ψ, θ, and p across sites (**S1 Table**). Of the five covariates included in the model (position coordinates X and Y, site depth, *C*. *tumulosa* abundance, and survey year), all were considered important predictors of *C*. *tumulosa* occupancy, with PIP scores greater than 0.5 (**S5 Table**). The abundance of *C*. *tumulosa* and the site depths were the most important predictors for qPCR replicate detection (p_11_). None of the included covariates were estimated to be important predictors of sample capture (θ). Modeled presence (which uses eDNA data, visual observation data, and habitat covariates) had no uncertainty if *C*. *tumulosa* was visually observed at a site (presence = 1, **Fig 3A**). However, when visual surveyors did not detect *C*. *tumulosa*, absence was correctly inferred at all but one site (K07) and there was some associated uncertainty in eDNA results from six sites (H01, H06, H07, KAP03, K18, and M04, **Fig 3A**). Modeled occupancy (which incorporates only habitat covariates) was non-zero for all sites, but was generally higher at sites from atolls where *C*. *tumulosa* was visually observed (Manawai, Kuaihelani, and Hōlanikū, **Fig 3B**). Inferring the absence of target eDNA was more uncertain at deeper sites (> 10 m, **Fig 4A and 4B**). Overall, occupancy was lower when *C*. *tumulosa* was visually absent (**Fig 4C**) and increased as *C*. *tumulosa* abundance increased (**Fig 4D**). The probability of eDNA sample capture (θ_11_) was consistent across sites (**Fig 4E and 4F**), whereas the probability of detection in qPCR replicates (p_11_) decreased with increasing water depth (**Fig 4G and 4H**).

**Fig 5:**
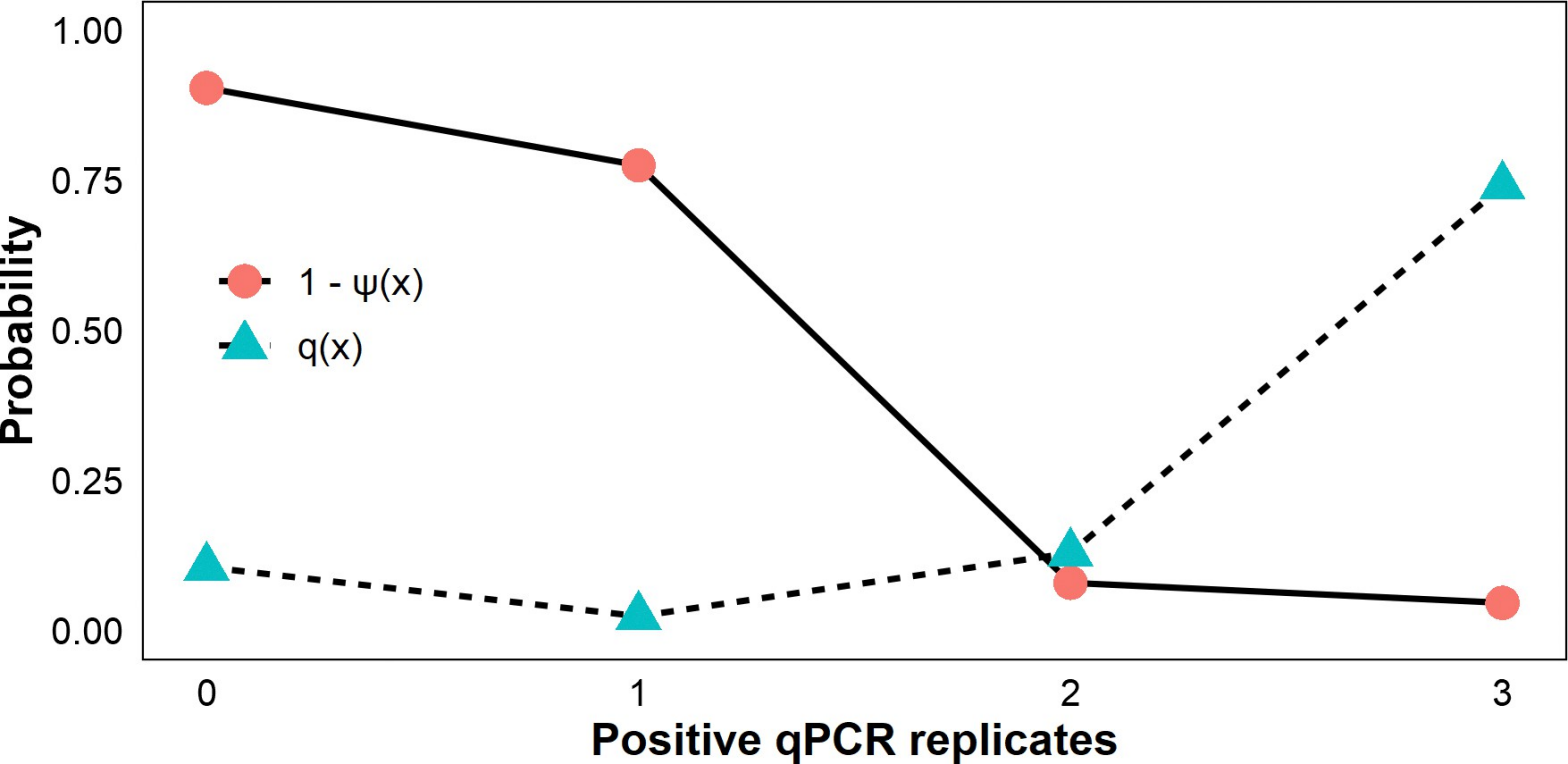
Estimation of qPCR replicate efficacy. Posterior conditional probability of target eDNA absence at a site given x quantitative polymerase chain reaction (qPCR) replicate positive amplifications, 1-ψ(x), and x positive qPCR replicates given target eDNA presence, q(x). Posterior conditional probabilities were estimated at the modal combination of the modeled covariates.

To gauge the performance of qPCR replication, we modeled the conditional posterior probability of species absence given x qPCR positive amplifications (i.e., the probability of generating a false-positive inference, 1-ψ(x)). There was a low probability of sites being unoccupied (< 8%) when two or more qPCR replicates amplify *C*. *tumulosa* eDNA (**Fig 5**). Additionally, given an occupied site, q(x), there was a combined 87% chance that two or more qPCR replicates amplified *C*. *tumulosa* eDNA using the assay.

### Estimation of eDNA errors

For sampling in the field, the baseline probability of true positive capture (i.e., a sample containing *C*. *tumulosa* eDNA from an occupied site, θ_11_) was estimated to be 0.89 (CI: 0.7–1.0, **S5 Table**). The probability of a false-negative test was estimated to be 0.11 (CI: 0.03–0.25) for field samples (1-θ_11_). The probability of false-positive inference (where non-eDNA methods estimate species absence but eDNA is present, θ_10_) was 0.03 (CI: 0–0.14).

In the lab, the baseline probability of true positive detection (i.e., a positive replicate from a sample containing *C*. *tumulosa* eDNA, p_11_) was estimated to be 0.94 (CI: 0.8–1.0), whereas the false-negative test error rate was estimated to be 0.06 (CI: 0.01–0.24) for qPCR replicates (1-p_11_). The probability of a false-positive test (i.e., contamination or PCR error, p_10_) was 0.02 (CI: 0.01–0.06).

Two individual technical-replicate equipment blanks from two separate sites (K09 and K14, **S9 Fig**) amplified *C*. *tumulosa* eDNA above the fluorescence quantification threshold, signifying sample contamination. However, this contamination was not evident in any other water samples from these sites and did not lead to false-positive inferences, therefore the corresponding sites were retained for the analyses. None of the NTC samples produced any signal amplification for *C*. *tumulosa*.

### Naïve occupancy

Naïve occupancy, determined from raw qPCR data, was 41% (26/63 sites). The assay detected *C*. *tumulosa* at 92% (24/26) of sites where its presence was visually known (**S9 Fig**). The two false-negative test sites were located at Hōlanikū (H08) and Kuaihelani (K10) which both exhibited ≤5% cover in visual surveys (**Fig 2**). At the other 21 sites where *C*. *tumulosa* was visually absent, raw qPCR results contained two false-positive inferences, one at Kuaihelani (K07) and Manawai (M04). No detections were apparent at sites where only eDNA was collected (OA01-OA16), and they are therefore presumed-negative.

**Fig 6:**
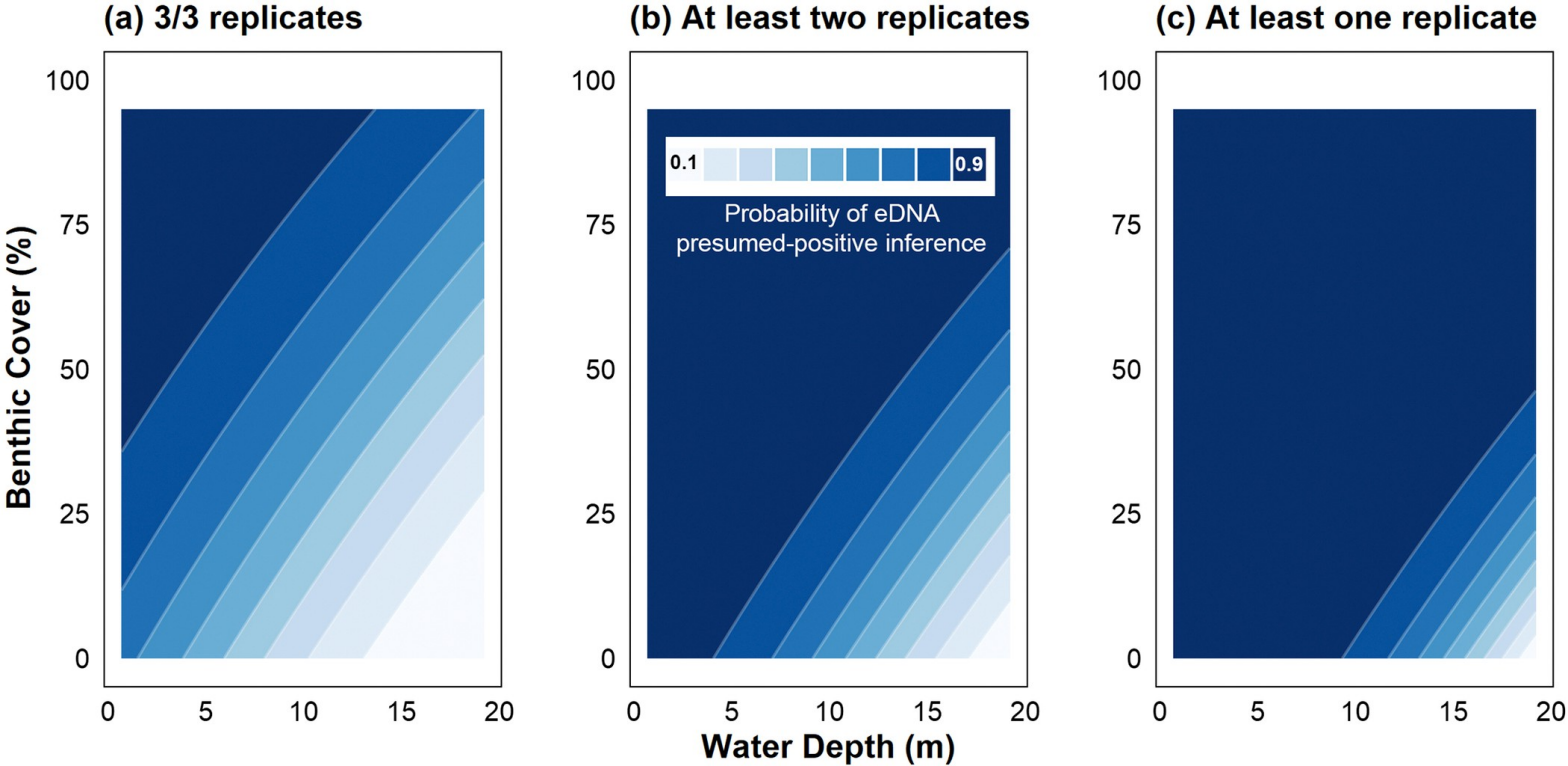
Assay detection probability surface under varying detection thresholds. Probability of inferring naïve *Chondria tumulosa* eDNA presence at a site with varying quantitative polymerase chain reaction (qPCR) detection thresholds: (a) a strict detection threshold of all three positive qPCR replicates, (b) a moderate threshold of at least two replicates amplifying, and (c) a lenient threshold where at least one of three replicates amplify. Surface smoothers were modeled using a beta regression of site-specific eDNA detection probabilities (p_11_) with color contours (bins = 9) corresponding to the likelihood of a presumed-positive inference across site depth and visual benthic cover of *C*. *tumulosa*.

The probability of detecting *C*. *tumulosa* in a qPCR replicate from samples containing eDNA (p_11_) was used to model the effects of site depth and abundance on naïve detection thresholds to infer presence-absence. Increasing water depth and decreasing benthic cover had an additive effect on positive qPCR detection of *C*. *tumulosa* (**Fig 6**). Using a strict detection threshold, requiring amplification of all three technical qPCR replicates for positive assignment, decreased the overall sensitivity across depths and abundances. Implementing a lenient threshold, where amplification of any positive qPCR replicates was required for detection, increased the likelihood of inferring *C*. *tumulosa* presence from surface seawater, particularly from shallow sites and those exhibiting extremely low benthic cover (< 1%).

### eDNA sequencing

Sequence data from field samples verified qPCR sensitivity and specificity. Most (96%) DNA reads matched 17 *C*. *tumulosa* MOTUs; the remaining reads matched another red alga in the tribe Polysiphonieae (Rhodomelaceae, Rhodophyta: GenBank ID OQ731395, 97.5% identity), primarily at sites lacking *C*. *tumulosa* (K16 and K18, **S10 Fig**), yet their amplification was not sufficient to create qPCR false-positive inferences.

## Discussion

*Chondria tumulosa* was visually observed at multiple locations at Hōlanikū, Kuaihelani, and Manawai atolls in PMNM. We incorporated this presence-absence data into site-occupancy models to predict the presence of target eDNA (i.e., is the target DNA there?) and generate a posterior probability of occupancy based on habitat covariates (i.e., do we expect the target DNA to be there?). There is a low probability that *C*. *tumulosa* is present outside of the atolls where it has been directly observed (Hōlanikū, Kuaihelani, and Manawai); however, there remains a non-zero chance of occupancy, a result driven by modeled site covariates: position coordinates, depth, and *C*. *tumulosa* abundance. For the time being, atolls southeast of *C*. *tumulosa*’s current distribution may be less at immediate risk, due to physical oceanographic barriers to migration between Manawai and Kapou, as indicated by ecological partitions [75], population genetic breaks [76, 77], and predominant surface currents moving up the island chain to the northwest [78, 79]. That said, Kapou remains the focus of both visual and eDNA surveillance, as it may represent a key colonization gateway to the rest of the archipelago (**Fig 2**). Molecular surveillance of ports and harbors may also be instrumental for early detection [80, 81], as future incursions are expected to occur through vegetative fragmentation [82] and hitchhiking on debris and vessels.

Our modeling of *C*. *tumulosa*’s distribution throughout PMNM provides a framework for interpreting qPCR results and enhancing field detection strategies at new sites, especially when the target organism cannot be visually detected. In general, site-occupancy detection modeling matched *C*. *tumulosa* presence-absence estimates from visual survey data and provided additional estimates of associated uncertainty regarding the assay’s use. Surface seawater sample collections captured *C*. *tumulosa* eDNA with high efficiency (89% mean capture rate) and qPCR replicates detected target eDNA with high sensitivity (94% mean detection rate). Target eDNA was detected at all three atolls where *C*. *tumulosa* was visually observed. The surface seawater assay is most effective at detecting *C*. *tumulosa* eDNA when site depths are shallow (< 10 m), likely due to stratification of eDNA at deeper sites [93, 99]. We suggest at least three qPCR technical replicates be analyzed from multiple sampling locations within a region and all available opportunistic visual data be incorporated into the model. False-negative test errors (*C*. *tumulosa* eDNA absent even though it was visually present) were 11% at the field sampling stage and 6% at the laboratory analysis stage. In contrast, the probability of a false-positive inference (*C*. *tumulosa* eDNA present even though it was visually absent) was 3% during field sampling and 2% during laboratory analysis. Our results emphasize the challenge of developing eDNA sampling strategies for nuisance species when their density and distributions are unknown.

### Estimation of eDNA errors

When comparing eDNA to visual surveys, the assay produced some apparent (site-level) false-positive inferences and (sample-level) false-negative tests (**S2 Fig**). Site-occupancy detection modeling estimated false-positive inference (≤ 3%) and false-negative test (≤ 11%) errors at both the field sample collection and lab qPCR replication stages (**S5 Table**). A first source of false-positive tests could be contamination, a crucial concern in molecular-based analyses [23]. However, we monitored contamination at several steps using appropriate controls and found minimal evidence of contamination that could create an unambiguous false-positive test. Second, non-specific amplification can create false-positive test detections. DNA sequencing of qPCR products did reveal a potential off-target species match (family Rhodomelaceae, tribe Polysiphonieae) which amplified at some sites in the absence of *C*. *tumulosa* (**S10 Fig**). While amplification of non-target DNA did not contribute to overall false-positive inferences based on visualization of qPCR data (**S9 Fig**), it did lead to ambiguities in eDNA modeling of presence-absence (**Fig 3A**), which we discuss below. Visual misclassification of sites is a third potential source of error. Visual benthic surveys have observer biases, but can also have errors from low abundance or misidentification, depending on the biology and ecology of the target species [34, 83]. Given *C*. *tumulosa*’s ability to grow cryptically, visual misidentifications could explain both false-positive inferences (e.g., by overlooking the target taxa) and false-negative eDNA detections (e.g., by misidentifying the target taxa for species such as *Palisada parvipapillata* or *Laurencia* spp.).

False-positive inferences can occur due to target eDNA being present even though individuals are not [84–87]. Rapid degradation of eDNA in oligotrophic, tropical marine environments resulting from site-specific factors such as sunlight, temperature, pH, and microbial activity suggests localized eDNA persistence with high site fidelity [87–97]. In the oligotrophic waters of PMNM, we expect high eDNA turnover with false-positive inferences being most influenced by surface rafting individuals (of a species susceptible to vegetative fragmentation) or, perhaps to a lesser degree, transport of eDNA in surface currents. Indeed, the false-positive inference at K07 could be explained by the visual presence of *C*. *tumulosa* at three adjacent sites (K02, K10, K11, **Fig 2**). Likewise, at M04, eDNA may be transported from adjacent sites with a high abundance of *C*. *tumulosa*; remote sensing with satellite imagery confirmed its presence nearby during the same time period [54]. All things considered, eDNA persistence in the water column (originating beyond our visually-surveyed 10 m radius) is the most likely explanation for observed false-positive inferences in this study. The limited number of false-positive inferences coupled with infrequent false-positive test errors implies that, when compared to visual surveys, the eDNA assay is robust to *C*. *tumulosa* presence-absence.

For nuisance species management, false-positive inferences may be vital for detecting inconspicuous life stages (e.g., larvae, spores, fragments) that may otherwise be overlooked. In this study, false-positive test errors were infrequent (2%). However, positive qPCR detection from a sample containing target eDNA (p_11_) is conflated with two possible causes: target eDNA is present in the sample due to the site being occupied (θ_11_) or target eDNA is present although the site is unoccupied (θ_10_). In the latter case, it becomes difficult to discriminate between detections due to eDNA persistence in the environment, field sample contamination, or non-specific amplification of off-target sequences. Visualization of qPCR amplification and melt curves (which can identify non-specific amplification, **S11 Fig**), negative controls at the sample level (such as field equipment blanks which can monitor contamination), and validation of amplicons with DNA sequencing (which can confirm PCR specificity) are therefore crucial to elucidate differences between errant site-level and sample-level detections. Encountering false-positives is unavoidable in biomonitoring, even with highly precise tests, particularly when population abundances are presumed to be very low [30]. When monitoring nuisance species in low abundance, users should be aware of the false-positive paradox [29], where the likelihood of a false-positive detection becomes greater than a true-positive detection due to target organisms being extremely rare.

Other potential pitfalls for interpretation of eDNA detection data come from false-negative test detections and ambiguous results. In this study, site-occupancy detection modeling provided vital estimates of uncertainty regarding the assay’s use, but modeled eDNA presence was discordant with expert visual observations of *C*. *tumulosa* at seven sites, six of which were ambiguous (with posterior credible intervals including both zero and one, **Fig 3A**). Visualization of raw qPCR data and sequencing of PCR products assisted presence-absence estimation where model results were ambiguous. For example, ambiguity of the result at K18 was resolved by sequencing that revealed non-specific amplification of a related alga in the absence of *C*. *tumulosa*, even though primer design *in silico* did not. Sequencing data also confirmed the presence of *C*. *tumulosa* eDNA at site M04 and K07, even though visual surveyors marked them as absent. As previously discussed, transport (of algal fragments or eDNA) in the water column is the most likely cause of false-positive inferences at these sites, although visual observation errors cannot be ruled out. The remaining ambiguous results from Hōlanikū (H01, H06, H07) and Kapou (KAP03, **Fig 3A**) could be attributed to concentrations of *C*. *tumulosa* eDNA lower than the limit of detection for this assay, with site depths (13.7–19.2 m) potentially contributing to eDNA dilution.

In terms of false-negative errors, there were two cases (Kuaihelani, K10 and Hōlanikū, H08) where visualization of raw qPCR data suggested false-negative test errors (**S2 Fig**). Occupancy modeling suggested that these marginally positive eDNA signals (with a single qPCR replicate amplifying) most likely reflect the presence of *C*. *tumulosa*, which was also confirmed by DNA sequencing (**S3 Fig**). One plausible explanation for low amplification fluorescence at these sites could be *in situ* dilution of target eDNA (Collins et al. 2018): H08 and K10 were the deepest sites (4.6 m) among those with relatively low benthic cover (≤ 5%). The biomass of target organisms and sampling distance from them are key predictors in eDNA detection [98–101]. Yet, statistical modeling was able to infer *C*. *tumulosa* presence at the deepest occupied site (M02, 18.6 m) and at multiple sites where cover was minimal (< 1%). In general, ambiguous inferences and false-negatives from surface samples were only observed when site depths exceeded 10 m (**Fig 4A**). However, modeled sample capture was consistent across depths and *C*. *tumulosa* abundances. Our results indicate that the field sample collection and extraction protocol is adequately capturing eDNA across depths, but the qPCR protocol is more likely to produce false-negatives as site depths increase. Ambiguous detections and false-negatives can presumably be further mitigated by limiting sampling to shallow sites (< 10 m) or collecting eDNA at depth through various means.

Uncertainty and errors in eDNA surveying are substantial barriers to biomonitoring adoption, triggering economic and social consequences when they are misrepresented [86]. Ideally, assays for early detection of nuisance species should have minimal associated errors. Our assay has higher false-negative than false-positive error rates. Therefore, validation of presumed-negative sites (e.g., through temporal sampling or direct observation) should be prioritized when species absence, rather than presence, is vital for decision-making—such as confirming eradication success. One fundamental assumption of site-occupancy detection models is that a site remains ‘closed’, meaning there are no changes to occupancy status (presence-absence) during surveillance of a site [42]. In other words, if a site was occupied by *C*. *tumulosa*, it was assumed to have been occupied throughout replicate water sample collection, and any failure to detect it is therefore considered a false-negative. Given that eDNA persists in seawater on the order of hours to days [93, 102], the closure assumption is likely met when using eDNA [36]. In occupancy modeling, the response variable aligns naturally with the sampling method used [103]. In this study, data are based on surveys conducted in a 10 m radius at each site; in theory, estimated presence signifies target eDNA presence within that specific area. In reality, site-occupancy modeling may indicate presence at a site, yet careful consideration must be made to address potential origins of target eDNA. Presumed-positive detections and suspected false-positive inferences can spur more intensive non-molecular surveillance [104], as the model explicitly estimates eDNA presence, not species presence. False-negative tests can be mitigated by maximizing eDNA capture and detection, for instance by filtering large volumes of water [39, 105]. Strict protocols must be followed by eDNA end users to prevent and account for sample contamination, for example through the use of multiple negative controls (e.g., equipment blanks and PCR controls), dedicated laboratory spaces, and estimation of error rates [22, 23, 106].

### Assay implementation

The capability to quickly and reliably gather extensive distribution data on a large scale is a key advantage of eDNA over traditional surveillance methods. Surveying in remote locations such as PMNM necessitates a careful balance between the number of sites or samples and the available resources, ensuring that data collection is both comprehensive yet feasible given logistical constraints. We developed the assay for use in monitoring programs with limited resources, implementing moderate biological sample replication (2–3 per site) and qPCR replication (3 per sample). To assess presence-absence discrimination among our three technical replicates, we estimated the conditional probability of eDNA presence given positive amplification of replicates. The probability of *C*. *tumulosa* eDNA presence becomes increasingly likely as the number of positive qPCR replicates increases: with two positive replicates the conditional probability of eDNA presence was 92% and with three positive replicates it was 95%. Therefore, using of two or more qPCR replicates is sufficient for detecting *C*. *tumulosa* eDNA with at least 92% certainty from surface seawater samples. With limited resources, maximizing the number of biological replicates (i.e., field samples) rather than technical replicates is advantageous [36, 107, 108].

Our assay is sensitive to *C*. *tumulosa* eDNA but is not be regarded as a standalone replacement for direct observations. The few discrepancies found between visual surveys and eDNA sampling in this study highlight the significance of obtaining direct observations whenever feasible, which likely has implications for biomonitoring elsewhere. For instance, our visual surveys were conducted by experts trained in identification of *C*. *tumulosa*. Utilizing non-specialist observers would presumably decrease the certainty of occupancy model estimates. However, if unambiguous detections are possible at some sites, this extra information can enhance the reliability of inferences [48]. Unambiguous detections can be achieved through visual surveys, but also through remote-sensing methods [54, 109] or by confirming ambiguous detections with high-throughput sequencing of PCR products. To this end, eDNA end users can balance the difficulties associated with visually surveying remote locations.

Biomonitoring in sensitive, remote locations may require generating occupancy results in near real-time to guide management efforts. Naïve estimates that do not incorporate modeling have been employed for this purpose, for example during rapid monitoring using portable qPCR machines *in situ* [110–112]. However, subjective selection of qPCR detection thresholds can impact the presumed-presence of target species [113–115]. By altering thresholds for presumed-positive inferences, our modeling of site characteristics suggests a tradeoff between sensitivity and reliability of our qPCR assay using surface seawater samples (**Fig 6**). On the one hand, implementing a strict threshold requiring all three technical replicates to amplify in order to infer naïve occupancy caused sensitivity to wane across all site depths and abundances; presumed-positive inference was 33% likely at moderate site depths (10 m) and low benthic cover (10%). On the other hand, requiring amplification of any single technical replicate increased overall detection sensitivity (with 92% likelihood at 10 m and 10% cover), but could be confounded by false-positive errors due to the high probability of species absence, 1- ψ(x), when only one qPCR replicate amplifies (**Fig 5**). Therefore, when occupancy is inferred from raw qPCR data, we suggest using a two-thirds majority threshold to balance sensitivity with false-positive inferences and false-positive test errors. In this way, the developed assay can be used to rapidly assess naïve presence-absence of *C*. *tumulosa* with probabilities exceeding 90% in water < 5 m (15 ft), especially at abundances far too low for reliable detection by an untrained visual observer.

## Conclusion

eDNA analyses are powerful tools for managing nuisance species [12, 19, 23, 26, 31] and can be enhanced by site-occupancy detection models [15, 51]. Statistical modeling of eDNA occupancy is vital for inferring presence-absence, reducing the risk that eDNA methods fail to detect nuisance species, and quantifying detection error rates across datasets [37, 38, 107]. Therefore site-occupancy detection models are necessary in any eDNA analyses for monitoring species presence. Based on the validation steps we’ve undertaken, our eDNA assay can be classified as “operational for routine monitoring” [25] (**S6 Table**). Our results provide strong evidence the assay can be used to presume *C*. *tumulosa* presence; confidence is somewhat reduced when presuming its absence, which may be required following eradication [6]. In general, eDNA analyses provide a marginal benefit where target taxa are in high abundance or where existing survey efforts are well-established [15]. Therefore, eDNA surveys are well-suited for biomonitoring of *C*. *tumulosa* across the Hawaiian Archipelago, especially where abundances are low (e.g., across its colonization front). Sampling for eDNA at potential source locations, such as in the remote understudied portions of the Pacific Ocean, may also resolve the cryptogenic status of *C*. *tumulosa*. Regular eDNA surveys are recommended to document the spread of nuisance species, resolve the alga’s cryptogenic status, and protect the biodiversity and functioning of threatened reefs.

Our eDNA assay builds upon previous work to adapt occupancy modeling to detect nuisance taxa, offering a reliable, sensitive, and cost-effective alternative to visual surveys. Imperfect detections can be incorporated into hierarchical occupancy frameworks, inferring presence-absence with objective estimates of uncertainty and allowing effective use of eDNA methods by partners without specialized skills in morphological identification. Using *C*. *tumulosa* in PMNM as a model system, we demonstrate the sensitivity of eDNA surveillance on coral reefs, highlighting potential sources of uncertainty for conservation management decision-making. Understanding the ecology of the target species is crucial for determining an optimal sampling strategy for detection with eDNA. Further efforts are still required to understand how life history traits and modes of reproduction [82] affect *C*. *tumulosa*’s eDNA persistence in the environment. Occupancy modeling of eDNA detections can be integrated into existing monitoring frameworks to further substantiate remote sensing data, focus preventative efforts, and map future range expansions of nuisance taxa.

## Supporting information

S1 Table Sampling locations for surface water collections of Chondria tumulosa environmental DNA (eDNA). Sites were spread across Papahānaumokuākea Marine National Monument (PMNM) and Oʻahu. Biological replicates (water samples, n = 160) varied due to logistical constraints on expeditions. The number of triplicate qPCR positive detections per sample at each site are listed. The posterior probability (and 95% credible intervals, CI) of eDNA presence, occupancy (ψ), sample capture given site presence (θ_11_), and detection probability given eDNA presence in a sample (p_11_) were estimated using an eDNA model incorporating false-positives and augmented visual survey data.
(DOCX)

S2 Table Primer site binding mismatches for species-specific C. tumulosa primers. Species-specific primer binding site mismatches for the chloroplast ribulose bisphosphate carboxylase large chain (rbcL) gene designed to exclusively amplify DNA from *Chondria tumulosa* (GenBank ID: MT039604). Target templates were matched *in silico* using the National Center for Biotechnology Information (NCBI) nucleotide collection (nt) Primer-BLAST tool (https://www.ncbi.nlm.nih.gov/tools/primer-blast/) or *in situ* using high-throughput sequencing of environmental DNA from water samples. Primer binding site mismatches are highlighted.
(DOCX)

S3 Table Assay reaction volumes and amplification details. Quantitative PCR (qPCR) reaction and thermal cycling parameters used in the assay.
(DOCX)

S4 Table Synthetic DNA fragments for standard curve dilution series. Synthetic double-stranded gBlocks® DNA fragment (Integrated DNA Technologies, Coralville, IA) used in the generation of a standard curve dilution series. The sequence from *Chondria tumulosa* (Accession: MT039604) is bolded with the additional CG-rich flanking regions (31 bp) on each end.
(DOCX)

S5 Table Site-occupancy detection model estimates and habitat covariates. Posterior mean and 95% credible interval (CI) for model regression coefficients: probability of eDNA site occupancy (ψ), true capture (θ_11_), false-positive inference capture (θ_10_), true detection (p_11_), and false-positive test detection (p_10_). Posterior inclusion probabilities (PIP, or proportion of iterations included in the model) of covariates linked to each parameter are also included. Model covariates included visual benthic cover, site depth, site position (X-Y coordinates), sample year, and the interaction of benthic cover and site depth (Cover:Depth) and position coordinates (X:Y). Coefficients with CIs that do not overlap zero and model covariates with PIP > 0.5 (the threshold applied to the most important model predictors, [38]), are bolded. The probability of absence (1-ψ), capture false-negative (1-θ_11_), capture true-negative (1-θ_10_), false-negative detection (1-p_11_), and true-negative detection (1-p_10_) are the complements of ψ, θ_11_, θ_10_, p_11_, and p_10_, respectively.
(DOCX)

S6 Table Assay readiness checklist. Readiness checklist and validation steps from Thalinger et al. [25] for the *Chondria tumulosa* quantitative polymerase chain reaction (qPCR) assay using environmental DNA.
(DOCX)

S1 Fig Assay validation with temperature gradient and standard curve. Assay development consisted of a temperature gradient approach on standard samples of DNA extracted from *Chondria tumulosa* tissue and no-template controls (NTC) to monitor for contamination. (a) The ideal annealing temperature (54.7–64°C) was selected among triplicate amplifications that produced a sigmoidal curve in the fewest cycles. (b) Melt curves were verified to only produce a single tall, narrow peak. (c) Threshold cycle (C_q_) values are plotted against triplicate DNA serial dilution starting concentrations to generate a best fit standard curve (R^2^ = 0.999, slope = -3.79, intercept = 33.62), using the curve-fitting approach [63]. Synthetic *C*. *tumulosa* DNA was quantified using a fluorometer (Qubit, Invitrogen) and diluted to create 10-fold serial dilutions ranging from 10^6^ to 10^0^ copies per reaction. The limit of detection (LOD, where 95% of technical replicates amplify) is marked with a solid red line and the limit of quantification (LOQ, the lowest initial DNA concentration quantifiable with a coefficient of variation below 35%) is marked with a dashed line.
(DOCX)

S2 Fig Schematic representation of eDNA site-occupancy model framework. Adapted from Griffin et al. [38] of the hierarchical site-occupancy model implemented in the RShiny application [66]. Environmental DNA is collected from *S* independent sites using *M* independent water samples, analyzed using *K* independent quantitative polymerase chain reactions (qPCR) replicates. Incidental observations confirming target species presence is denoted as *k* with a common probability of confirmed detection, *π*. For every *s*th site and *m*th water sample, a positive qPCR result is denoted as *y*_*sm*_. The occupancy state defines eDNA presence-absence at a site, *z*, or in a sample, *w*. The probability of eDNA being present at a site, *ѱ*, captured in a sample, *θ*, or detected in an individual qPCR replicate, *p*, are estimated. More specifically, the model estimates field (stage 1) capture using the probability of eDNA presence in a sample from a site is *θ*_*11*_ if the site was occupied and *θ*_*10*_ if it was unoccupied (with false-negatives and true negatives being *1-θ*_*11*_ and *1-θ*_*10*_, respectively). Laboratory (stage 2) detection is estimated with positive qPCR replicates from samples containing target eDNA, *p*_*11*_, or not containing target eDNA, *p*_*10*_ (with false negatives and true negatives being *1-p*_*11*_ and *1-p*_*10*_, respectively).
(DOCX)

S3 Fig Posterior summary of occupancy. Posterior summaries of the probability of baseline environmental DNA (eDNA) occupancy at a site (ψ) resulting from site-occupancy detection modeling using the RShiny application.
(DOCX)

S4 Fig Posterior summary of capture. Posterior summaries of the probability of baseline environmental DNA (eDNA) sample capture given presence of target eDNA at a site (θ_11_) resulting from site-occupancy detection modeling using the RShiny application.
(DOCX)

S5 Fig Posterior summary of detection. Posterior summaries of the probability of baseline environmental DNA (eDNA) qPCR replicate detection given presence of target eDNA in a sample (p_11_) resulting from site-occupancy detection modeling using the RShiny application.
(DOCX)

S6 Fig Trace plot of occupancy. Environmental DNA (eDNA) probability of site occupancy (ψ) model fit trace plot, effective sample sizes (ESS), and Geweke diagnostics from eDNA site-occupancy detection modeling using the RShiny application.
(DOCX)

S7 Fig Trace plot of capture. Environmental DNA (eDNA) sample capture given presence of target eDNA at a site (θ_11_) model fit trace plot, effective sample sizes (ESS), and Geweke diagnostics from eDNA site-occupancy detection modeling using the RShiny application.
(DOCX)

S8 Fig Trace plot of detection. Environmental DNA (eDNA) qPCR replicate detection given presence of target eDNA in a sample (p_11_) model fit trace plot, effective sample sizes (ESS), and Geweke diagnostics from eDNA site-occupancy detection modeling using the RShiny application.
(DOCX)

S9 Fig Amplification curves for surveyed sites across the Hawaiian Archipelago. Environmental DNA (eDNA) quantitative polymerase chain reaction (qPCR) amplification curves and visual benthic categorization from across the Hawaiian Archipelago. Sites with corresponding visual data (H01 through L04) were from Hōlanikū (“H”, or Kure Atoll), Kuaihelani (“K”, or Midway Island), Manawai (“M”, or Pearl & Hermes Atoll), Kapou (“KAP”, or Lisianski Island), and Lalo (“L”, or French Frigate Shoals). Sites lacking visual data (OA01 through OA17) were from Oʻahu (“OA”). Amplification (relative fluorescence units, RFU) of *Chondria tumulosa* eDNA is marked with circles (representing each individual PCR replicate) and a solid line generalized additive model smoother of triplicate PCR reactions among water samples from each site. Line colors refer to the visual categorization of sites from visual surveys (green: “Absent”, orange: “Present”). Control samples, consisting of positive *C*. *tumulosa* tissue extractions (“Standard”), equipment blanks (“EB”), and qPCR no-template controls (“NTC”) are marked in black. The mean fluorescence quantification threshold is marked with a dashed grey line. The standard error of the mean (± SE) is shaded, but is often so narrow that it becomes obscured by the curve.
(DOCX)

S10 Fig Normalized eDNA reads from high-throughput sequencing of samples. Environmental DNA index (normalized using the inversed order of operations of the Wisconsin double-standardization) from molecular operational taxonomic units (MOTUs) that were amplified and sequenced using the assay. Amplicons were sequenced on an Illumina MiSeq platform and matched with NCBI’s BLASTn algorithm (%ID > 97, E-value < 1e-10). *Chondria tumulosa* matched 17 MOTUs and six MOTUs matched to the family Rhodomelaceae, tribe Polysiphonieae. Bar colors refer to the categorization of *C*. *tumulosa* from visually surveyed sites (green: “Absent”, orange: “Present”). Samples were from Kuaihelani (“K”, or Midway Island), Manawai (“M”, or Pearl & Hermes Atoll), or Oʻahu (“OA”).
(DOCX)

S11 Fig Validation of qPCR amplification and melt curves. Example validation of positive detections using quantitative polymerase chain reaction (qPCR) (a) amplification (relative fluorescence units, RFU) curves and (b) melt curve analysis. Panels depict DNA extracted from positive control *Chondria tumulosa* tissue (Standard, n = 39), no-template controls (NTC, n = 39), equipment blanks (“EB”, n = 63), an exemplar field positive detection at Kuaihelani (K17), and an ambiguous field site which amplified DNA from an unknown alga in the tribe Polysiphonieae (K18). The mean (± standard error of the mean) fluorescence quantification threshold is marked with a dashed black line. Low-level contamination was detected in EB samples from two sites at Kuaihelani (K09 & K14); each had a single technical qPCR replicate amplify above the fluorescence threshold.
(DOCX)
