# Supplementary material for: Navigating uncertainty in environmental DNA detection of a nuisance marine macroalga
Source: PLoS One. 2025 Feb 4;20(2):e0318414. doi: 10.1371/journal.pone.0318414 (PMC11793909; doi:10.1371/journal.pone.0318414)
Supplement: S1 Table — Sites were spread across Papahānaumokuākea Marine National Monument (PMNM) and Oʻahu. Biological replicates (water samples, n = 160) varied due to logistical constraints on expeditions. The number of triplicate qPCR positive detections per sample at each site are listed. The posterior probability (and 95% credible intervals, CI) of eDNA presence, occupancy (ψ), sample capture given site presence (θ11), and detection probability given eDNA presence in a sample (p11) were estimated using an eDNA model incorporating false-positives and augmented visual survey data. (DOCX) [file pone.0318414.s001.docx]

**S1 Table. Sampling locations for surface water collections of *Chondria tumulosa* environmental DNA (eDNA).** Sites were spread across Papahānaumokuākea Marine National Monument (PMNM) and Oʻahu. Biological replicates (water samples, n=160) varied due to logistical constraints on expeditions. The number of triplicate qPCR positive detections per sample at each site are listed. The posterior probability (and 95% credible intervals, CI) of eDNA presence, occupancy (ψ), sample capture given site presence (θ_11_), and detection probability given eDNA presence in a sample (p_11_) were estimated using an eDNA model incorporating false-positives and augmented visual survey data.

| **Sites** | **Date** | **Island** | **Depth (m)** | **% Cover** | **Lat.** | **Long.** | **Samples** | **PCR detection** | **Presence (CI)** | **Occupancy** ψ **(CI)** | **θ_11_ (CI)** | **p_11_ (CI)** |
| --- | --- | --- | --- | --- | --- | --- | --- | --- | --- | --- | --- | --- |
| H01 | 7/11/2023 | Hōlanikū | 14.9 | 0 | 28.40905 | -178.378 | 2 | 1, 1 | 0.32 (0,1) | 0.44 (0.1, 0.8) | 0.86 (0.6, 1) | 0.39 (0.1, 0.9) |
| H02 | 7/11/2023 | Hōlanikū | 1.8 | <1 | 28.41977 | -178.3719 | 2 | 3, 3 | 1 | 0.60 (0.4, 0.8) | 0.87 (0.7, 1) | 0.94 (0.8, 1) |
| H03 | 7/11/2023 | Hōlanikū | 2.9 | <1 | 28.42866 | -178.36946 | 2 | 3, 1 | 1 | 0.59 (0.4, 0.8) | 0.87 (0.7, 1) | 0.92 (0.7, 1) |
| H04 | 7/11/2023 | Hōlanikū | 1.5 | <1 | 28.45352 | -178.32867 | 2 | 3, 1 | 1 | 0.61 (0.4, 0.8) | 0.87 (0.7, 1) | 0.94 (0.8, 1) |
| H05 | 7/11/2023 | Hōlanikū | 1.5 | <1 | 28.42157 | -178.2881 | 2 | 3, 3 | 1 | 0.60 (0.4, 0.8) | 0.87 (0.7, 1) | 0.94 (0.8, 1) |
| H06 | 7/11/2023 | Hōlanikū | 16.8 | 0 | 28.45377 | -178.34331 | 2 | 0, 0 | 0.06 (0,1) | 0.43 (0.1, 0.8) | 0.86 (0.6, 1) | 0.29 (0, 0.8) |
| H07 | 7/11/2023 | Hōlanikū | 13.7 | 0 | 28.38196 | -178.32465 | 2 | 0, 0 | 0.03 (0,1) | 0.45 (0.1, 0.8) | 0.86 (0.6, 1) | 0.45 (0.1, 0.9) |
| H08 | 7/12/2023 | Hōlanikū | 4.6 | <1 | 28.44074 | -178.36086 | 2 | 0, 2 | 1 | 0.57 (0.3, 0.8) | 0.87 (0.7, 1) | 0.88 (0.6, 1) |
| H09 | 7/12/2023 | Hōlanikū | 1.8 | 0 | 28.38854 | -178.3165 | 2 | 1, 0 | 0.01 | 0.60 (0.4, 0.8) | 0.87 (0.7, 1) | 0.94 (0.8, 1) |
| H10 | 7/12/2023 | Hōlanikū | 0.9 | 0 | 28.39353 | -178.29643 | 2 | 0, 0 | 0.01 | 0.61 (0.4, 0.8) | 0.87 (0.7, 1) | 0.95 (0.8, 1) |
| K01 | 7/19/2022 | Kuaihelani | 3.7 | 5 | 28.25624 | -177.37148 | 3 | 3, 3, 3 | 1 | 0.64 (0.5, 0.8) | 0.88 (0.8, 1) | 0.95 (0.9, 1) |
| K02 | 7/20/2022 | Kuaihelani | 3.7 | 1 | 28.21831 | -177.4134 | 3 | 3, 3, 3 | 1 | 0.61 (0.4, 0.8) | 0.88 (0.8, 1) | 0.94 (0.8, 1) |
| K03 | 7/23/2022 | Kuaihelani | 1.2 | 95 | 28.24782 | -177.35305 | 3 | 0, 3, 3 | 1 | 0.93 (0.6, 1) | 0.88 (0.8, 1) | 1 |
| K04 | 7/23/2022 | Kuaihelani | 2.4 | 20 | 28.2534 | -177.35502 | 3 | 0, 3, 3 | 1 | 0.75 (0.6, 0.9) | 0.88 (0.8, 1) | 0.98 (0.9, 1) |
| K05 | 7/23/2022 | Kuaihelani | 1.5 | 85 | 28.2495 | -177.35526 | 3 | 3, 3, 3 | 1 | 0.92 (0.7, 1) | 0.88 (0.8, 1) | 1 |
| K06 | 7/19/2022 | Kuaihelani | 3.1 | 30 | 28.264546 | -177.39002 | 3 | 3, 3, 3 | 1 | 0.80 (0.6, 0.9) | 0.88 (0.8, 1) | 0.98 (0.9, 1) |
| K07 | 7/16/2022 | Kuaihelani | 2.7 | 0 | 28.228584 | -177.42071 | 3 | 3, 3, 3 | 1 | 0.62 (0.4, 0.8) | 0.88 (0.8, 1) | 0.95 (0.9, 1) |
| K08 | 7/17/2022 | Kuaihelani | 1.1 | 20 | 28.270209 | -177.34113 | 3 | 1, 3, 3 | 1 | 0.75 (0.6, 0.9) | 0.88 (0.8, 1) | 0.99 (0.9, 1) |
| K09 | 7/17/2022 | Kuaihelani | 1.4 | 75 | 28.259523 | -177.33237 | 3 | 3, 3, 3 | 1 | 0.91 (0.6, 1) | 0.88 (0.8, 1) | 1 |
| K10 | 7/16/2022 | Kuaihelani | 4.6 | 5 | 28.227209 | -177.4042 | 3 | 1, 1, 1 | 1 | 0.63 (0.4, 0.8) | 0.88 (0.8, 1) | 0.94 (0.8, 1) |
| K11 | 7/16/2022 | Kuaihelani | 2.7 | 1 | 28.222918 | -177.41577 | 3 | 3, 3, 3 | 1 | 0.62 (0.4, 0.8) | 0.88 (0.8, 1) | 0.96 (0.9, 1) |
| K12 | 7/15/2022 | Kuaihelani | 1.5 | 5 | 28.27735 | -177.36612 | 3 | 3, 3, 3 | 1 | 0.67 (0.5, 0.8) | 0.88 (0.8, 1) | 0.97 (0.9, 1) |
| K13 | 7/18/2022 | Kuaihelani | 2.7 | 80 | 28.243191 | -177.33725 | 3 | 3, 3, 3 | 1 | 0.92 (0.7, 1) | 0.88 (0.8, 1) | 0.99 (0.9, 1) |
| K14 | 7/18/2022 | Kuaihelani | 7.6 | 0 | 28.218221 | -177.36817 | 3 | 0, 0, 0 | 0 | 0.55 (0.3, 0.8) | 0.88 (0.8, 1) | 0.86 (0.6, 1) |
| K15 | 7/18/2022 | Kuaihelani | 1.8 | 0 | 28.214474 | -177.36302 | 3 | 0, 0, 0 | 0 | 0.63 (0.4, 0.8) | 0.88 (0.8, 1) | 0.96 (0.9, 1) |
| K16 | 7/18/2022 | Kuaihelani | 0.9 | 0 | 28.211504 | -177.36812 | 3 | 0, 0, 0 | 0 | 0.64 (0.4, 0.8) | 0.88 (0.8, 1) | 0.97 (0.9, 1) |
| K17 | 7/17/2022 | Kuaihelani | 1.1 | 20 | 28.265998 | -177.34147 | 3 | 3, 3, 3 | 1 | 0.76 (0.6, 0.9) | 0.88 (0.8, 1) | 0.99 (0.9, 1) |
| K18 | 7/20/2022 | Kuaihelani | 10.7 | 0 | 28.19637 | -177.375 | 3 | 2, 2, 0 | 0.63 (0,1) | 0.51 (0.2, 0.8) | 0.88 (0.7, 1) | 0.72 (0.4, 1) |
| K19 | 7/19/2022 | Kuaihelani | 1.8 | 5 | 28.26913 | -177.38635 | 3 | 3, 3, 3 | 1 | 0.66 (0.5, 0.8) | 0.88 (0.8, 1) | 0.97 (0.9, 1) |
| KAP01 | 7/9/2023 | Kapou | 10.1 | 0 | 26.00257 | -173.9646 | 2 | 0, 0 | 0.01 | 0.29 (0.1, 0.5) | 0.87 (0.6, 1) | 0.61 (0.1, 1) |
| KAP02 | 7/9/2023 | Kapou | 7.3 | 0 | 26.03954 | -174.00101 | 2 | 0, 0 | 0.01 | 0.32 (0.1, 0.5) | 0.87 (0.6, 1) | 0.74 (0.2, 1) |
| KAP03 | 7/9/2023 | Kapou | 19.2 | 0 | 26.07841 | -173.997 | 2 | 0, 0 | 0.03 (0,1) | 0.24 (0, 0.6) | 0.86 (0.5, 1) | 0.18 (0, 0.7) |
| KAP04 | 7/9/2023 | Kapou | 3.1 | 0 | 25.96734 | -173.91605 | 2 | 0, 0 | 0 | 0.36 (0.2, 0.6) | 0.87 (0.6, 1) | 0.88 (0.4, 1) |
| KAP05 | 7/9/2023 | Kapou | 4.9 | 0 | 26.06117 | -173.95456 | 2 | 0, 0 | 0 | 0.35 (0.2, 0.6) | 0.87 (0.6, 1) | 0.83 (0.3, 1) |
| L01 | 7/6/2023 | Lalo | 10.7 | 0 | 23.77066 | -166.26059 | 2 | 0, 0 | 0 | 0.11 (0, 0.3) | 0.86 (0.5, 1) | 0.51 (0, 1) |
| L02 | 7/6/2023 | Lalo | 6.1 | 0 | 23.86975 | -166.28839 | 2 | 0, 0 | 0 | 0.14 (0, 0.3) | 0.86 (0.5, 1) | 0.69 (0, 1) |
| L03 | 7/6/2023 | Lalo | 2.7 | 0 | 23.78871 | -166.20895 | 2 | 0, 0 | 0 | 0.16 (0, 0.4) | 0.86 (0.5, 1) | 0.79 (0.1, 1) |
| L04 | 7/6/2023 | Lalo | 6.1 | 0 | 23.74216 | -166.19913 | 2 | 0, 0 | 0 | 0.13 (0, 0.3) | 0.86 (0.5, 1) | 0.69 (0, 1) |
| M01 | 7/18/2021 | Manawai | 7.6 | 45 | 27.81857 | -175.98833 | 4 | 3, 2, 3, 3 | 1 | 0.83 (0.6, 1) | 0.89 (0.8, 1) | 0.97 (0.9, 1) |
| M02 | 7/14/2021 | Manawai | 18.6 | 40 | 27.79029 | -176.0001 | 4 | 1, 2, 1, 2 | 1 | 0.78 (0.3, 1) | 0.88 (0.7, 1) | 0.55 (0.3, 0.8) |
| M03 | 7/14/2021 | Manawai | 9.1 | 75 | 27.79041 | -175.99626 | 4 | 3, 3, 3, 3 | 1 | 0.92 (0.7, 1) | 0.89 (0.8, 1) | 0.98 (0.9, 1) |
| M04 | 7/15/2021 | Manawai | 12.2 | 0 | 27.78565 | -175.82392 | 4 | 3, 3, 1, 2 | 0.90 (0,1) | 0.44 (0.1, 0.7) | 0.88 (0.8, 1) | 0.67 (0.4, 0.9) |
| M05 | 7/16/2021 | Manawai | 7.6 | 75 | 27.94199 | -175.72786 | 4 | 3, 3, 3, 3 | 1 | 0.92 (0.7, 1) | 0.89 (0.8, 1) | 0.99 (0.9, 1) |
| M06 | 7/16/2021 | Manawai | 10.7 | 60 | 27.88029 | -175.72669 | 4 | 3, 3, 0, 0 | 1 | 0.89 (0.6, 1) | 0.89 (0.8, 1) | 0.95 (0.9, 1) |
| M07 | 7/16/2021 | Manawai | 13.4 | 55 | 27.85155 | -175.74023 | 1 | 3 | 1 | 0.87 (0.6, 1) | 0.89 (0.8, 1) | 0.89 (0.8, 1) |
| M08 | 7/18/2021 | Manawai | 6.4 | 70 | 27.89012 | -175.92347 | 4 | 3, 3, 3, 3 | 1 | 0.91 (0.7, 1) | 0.89 (0.8, 1) | 0.99 (0.9, 1) |
| M09 | 7/20/2021 | Manawai | 7.6 | 0 | 27.77585 | -175.9742 | 4 | 0, 0, 0, 0 | 0 | 0.50 (0.3, 0.7) | 0.89 (0.8, 1) | 0.88 (0.7, 1) |
| OA01 | 10/19/2022 | Oʻahu | 0.7 |  | 21.364043 | -157.951918 | 2 | 0, 0 | 0 | 0.06 (0, 0.2) | 0.82 (0.1, 1) | 0.74 (0, 1) |
| OA02 | 10/19/2022 | Oʻahu | 2.4 |  | 21.359793 | -157.958856 | 2 | 0, 0 | 0 | 0.06 (0, 0.2) | 0.82 (0.1, 1) | 0.71 (0, 1) |
| OA03 | 10/19/2022 | Oʻahu | 1 |  | 21.359365 | -157.968804 | 2 | 0, 0 | 0 | 0.06 (0, 0.2) | 0.82 (0.1, 1) | 0.73 (0, 1) |
| OA04 | 10/19/2022 | Oʻahu | 1.5 |  | 21.365703 | -157.966024 | 2 | 0, 0 | 0 | 0.06 (0, 0.2) | 0.82 (0.1, 1) | 0.72 (0, 1) |
| OA05 | 10/13/2022 | Oʻahu | 12 |  | 21.311866 | -157.875015 | 2 | 0, 0 | 0 | 0.04 (0, 0.2) | 0.82 (0.1, 1) | 0.46 (0, 1) |
| OA06 | 11/3/2022 | Oʻahu | 1.8 |  | 21.444034 | -157.809659 | 2 | 0, 0 | 0 | 0.06 (0, 0.2) | 0.82 (0.1, 1) | 0.72 (0, 1) |
| OA07 | 11/3/2022 | Oʻahu | 3 |  | 21.444101 | -157.810017 | 2 | 0, 0 | 0 | 0.06 (0, 0.2) | 0.82 (0.1, 1) | 0.69 (0, 1) |
| OA08 | 11/3/2022 | Oʻahu | 2 |  | 21.44322 | -157.809822 | 2 | 0, 0 | 0 | 0.06 (0, 0.2) | 0.82 (0.1, 1) | 0.72 (0, 1) |
| OA09 | 10/13/2022 | Oʻahu | 1.5 |  | 21.312267 | -157.889539 | 2 | 0, 0 | 0 | 0.06 (0, 0.2) | 0.82 (0.1, 1) | 0.72 (0, 1) |
| OA10 | 10/13/2022 | Oʻahu | 1 |  | 21.302482 | -157.869673 | 2 | 0, 0 | 0 | 0.06 (0, 0.2) | 0.82 (0.1, 1) | 0.73 (0, 1) |
| OA11 | 10/13/2022 | Oʻahu | 1 |  | 21.300897 | -157.876492 | 2 | 0, 0 | 0 | 0.06 (0, 0.2) | 0.82 (0.1, 1) | 0.73 (0, 1) |
| OA12 | 10/17/2022 | Oʻahu | 5.5 |  | 21.293013 | -157.85513 | 2 | 0, 0 | 0 | 0.05 (0, 0.2) | 0.82 (0.1, 1) | 0.64 (0, 1) |
| OA13 | 10/17/2022 | Oʻahu | 1 |  | 21.290907 | -157.859165 | 2 | 0, 0 | 0 | 0.06 (0, 0.2) | 0.82 (0.1, 1) | 0.73 (0, 1) |
| OA14 | 10/17/2022 | Oʻahu | 5.5 |  | 21.293116 | -157.858853 | 2 | 0, 0 | 0 | 0.05 (0, 0.2) | 0.82 (0.1, 1) | 0.65 (0, 1) |
| OA15 | 10/17/2022 | Oʻahu | 1.5 |  | 21.290521 | -157.860994 | 2 | 0, 0 | 0 | 0.06 (0, 0.2) | 0.82 (0.1, 1) | 0.72 (0, 1) |
| OA16 | 6/1/2021 | Oʻahu | 1 |  | 21.274375 | -157.76046 | 2 | 0, 0 | 0 | 0.06 (0, 0.2) | 0.83 (0.1, 1) | 0.75 (0, 1) |
