# Supplementary material for: Navigating uncertainty in environmental DNA detection of a nuisance marine macroalga
Source: PLoS One. 2025 Feb 4;20(2):e0318414. doi: 10.1371/journal.pone.0318414 (PMC11793909; doi:10.1371/journal.pone.0318414)
Supplement: S2 Table — Species-specific primer binding site mismatches for the chloroplast ribulose bisphosphate carboxylase large chain (rbcL) gene designed to exclusively amplify DNA from Chondria tumulosa (GenBank ID: MT039604). Target templates were matched in silico using the National Center for Biotechnology Information (NCBI) nucleotide collection (nt) Primer-BLAST tool (https://www.ncbi.nlm.nih.gov/tools/primer-blast/) or in situ using high-throughput sequencing of environmental DNA from water samples. Primer binding site mismatches are highlighted. (DOCX) [file pone.0318414.s002.docx]

**S2 Table. Primer site binding mismatches for species-specific *C. tumulosa* primers.** Species-specific primer binding site mismatches for the chloroplast ribulose bisphosphate carboxylase large chain (rbcL) gene designed to exclusively amplify DNA from *Chondria tumulosa* (GenBank ID: MT039604). Target templates were matched *in silico* using the National Center for Biotechnology Information (NCBI) nucleotide collection (nt) Primer-BLAST tool (<https://www.ncbi.nlm.nih.gov/tools/primer-blast/>) or *in situ* using high-throughput sequencing of environmental DNA from water samples. Primer binding site mismatches are highlighted.

| **Accession #** | **Organism** | **Forward (5’->3’)** | **Reverse (5’->3’)** | **Detected** |
| --- | --- | --- | --- | --- |
| MT039604 | *Chondria* *tumulosa* | GCCGTGAATCGTTCTATTGC | TCAGCTCTTTCGTACATATTCTCC | *In silico* |
| MT039602 | *Chondria* *tumulosa* | GCCGTGAATCGTTCTATTGC | TCAGCTCTTTCGTACATATTCTCC | *In silico* |
| MT039601 | *Chondria* *tumulosa* | GCCGTGAATCGTTCTATTGC | TCAGCTCTTTCGTACATATTCTCC | *In silico* |
| NC_057618 | *Chondria* *tumulosa* | GCCGTGAATCGTTCTATTGC | TCAGCTCTTTCGTACATATTCTCC | *In silico* |
| MT039606 | *Chondria* *tumulosa* | G**A**CGTGAATCGTTCTATTGC | TCAGCTCTTTCGTACATATTCTCC | *In silico* |
| OQ731395 | *Echinothamnion* sp. (Polysiphonieae) | GC**T**GT**A**AATCGTTC**A**ATTGC | TCAGCTCTTTCGTACATAT**CT**TCC | *In situ* |
| OK209862 | *Yuzurua* sp. | GCCGT**T**AATCGTTCTAT**A**GC | TCAGCTCTTTC**A**TACAT**G**T**CT**TCC | *In silico* |
