# Supplementary material for: Navigating uncertainty in environmental DNA detection of a nuisance marine macroalga
Source: PLoS One. 2025 Feb 4;20(2):e0318414. doi: 10.1371/journal.pone.0318414 (PMC11793909; doi:10.1371/journal.pone.0318414)
Supplement: S3 Table — Quantitative PCR (qPCR) reaction and thermal cycling parameters used in the assay. (DOCX) [file pone.0318414.s003.docx]

**S3 Table. Assay reaction volumes and amplification details**. Quantitative PCR (qPCR) reaction and thermal cycling parameters used in the assay.

| **Reagent** | **Volume (µL)** | **Notes** |
| --- | --- | --- |
| DNA-free water | 3 | Growcells, USA |
| Primer CTrbcL_F1 | 0.5 | 10 µM |
| Primer CTrbcL_R1 | 0.5 | 10 µM |
| SSoAdvanced SybrGreen Universal Supermix | 4.5 | Bio-Rad, USA |
| Bovine Serum Albumin (BSA) | 0.5 | ThermoFisher Scientific, USA, 20 mg mL-1 |
| Template DNA | 1 |  |
|  | | |
| **qPCR step** | **Temp. (°C)** | **Duration** |
| Initial denaturation | 95 | 3 min. |
| *10 cycles:* |  | |
| Denaturation | 95 | 30 s |
| Annealing | 67 | 30 s, decrease 1 °C per cycle |
| Extension | 72 | 30 s |
| *30 cycles:* |  | |
| Denaturation | 95 | 30 s |
| Annealing | 54-64 | 30 s |
| Extension | 72 | 30 s, fluorescence reading |
| Final Extension | 72 | 10 min. |
| Melt Curve Analysis | 65-95 | Fluorescence reading, increase 1 °C per cycle |
