# Supplementary material for: Navigating uncertainty in environmental DNA detection of a nuisance marine macroalga
Source: PLoS One. 2025 Feb 4;20(2):e0318414. doi: 10.1371/journal.pone.0318414 (PMC11793909; doi:10.1371/journal.pone.0318414)
Supplement: S4 Table — Synthetic double-stranded gBlocks® DNA fragment (Integrated DNA Technologies, Coralville, IA) used in the generation of a standard curve dilution series. The sequence from Chondria tumulosa (Accession: MT039604) is bolded with the additional CG-rich flanking regions (31 bp) on each end. (DOCX) [file pone.0318414.s004.docx]

**S4 Table. Synthetic DNA fragments for standard curve dilution series**. Synthetic double-stranded gBlocks® DNA fragment (Integrated DNA Technologies, Coralville, IA) used in the generation of a standard curve dilution series. The sequence from *Chondria tumulosa* (Accession: MT039604) is bolded with the additional CG-rich flanking regions (31 bp) on each end.

| Sequence (5’ - 3’) | Length (base pairs) | % GC | Molecular Weight |
| --- | --- | --- | --- |
| CCGGCCCGCCCGATTTTTATATTCAATGGAA**GCCGTGAATCGTTCTATTGCTGCAACTGGAGAAGTAAAAGGTCATTACATGAACGTAACAGCAGCAACTATGGAGAATATGTACGAAAGAGCTGA**ATTTGCAAAACAATTAGGCCCCGCCCCCCCC | 157 | 46.5 | 96880.7 |
