# Supplementary material for: Navigating uncertainty in environmental DNA detection of a nuisance marine macroalga
Source: PLoS One. 2025 Feb 4;20(2):e0318414. doi: 10.1371/journal.pone.0318414 (PMC11793909; doi:10.1371/journal.pone.0318414)
Supplement: S5 Table — Posterior mean and 95% credible interval (CI) for model regression coefficients: probability of eDNA site occupancy (ψ), true capture (θ11), false-positive inference capture (θ10), true detection (p11), and false-positive test detection (p10). Posterior inclusion probabilities (PIP, or proportion of iterations included in the model) of covariates linked to each parameter are also included. Model covariates included visual benthic cover, site depth, site position (X-Y coordinates), sample year, and the interaction of benthic cover and site depth (Cover:Depth) and position coordinates (X:Y). Coefficients with CIs that do not overlap zero and model covariates with PIP > 0.5 (the threshold applied to the most important model predictors, [38]), are bolded. The probability of absence (1-ψ), capture false-negative (1-θ11), capture true-negative (1-θ10), false-negative detection (1-p11), and true-negative detection (1-p10) are the complements of ψ, θ11, θ10, p11, and p10, respectively. (DOCX) [file pone.0318414.s005.docx]

**S5 Table. Site-occupancy detection model estimates and habitat covariates.** Posterior mean and 95% credible interval (CI) for model regression coefficients: probability of eDNA site occupancy (ψ), true capture (θ_11_), false-positive inference capture (θ_10_), true detection (p_11_), and false-positive test detection (p_10_). Posterior inclusion probabilities (PIP, or proportion of iterations included in the model) of covariates linked to each parameter are also included. Model covariates included visual benthic cover, site depth, site position (X-Y coordinates), sample year, and the interaction of benthic cover and site depth (Cover:Depth) and position coordinates (X:Y). Coefficients with CIs that do not overlap zero and model covariates with PIP > 0.5 (the threshold applied to the most important model predictors, [38]), are bolded. The probability of absence (1-ψ), capture false-negative (1-θ_11_), capture true-negative (1-θ_10_), false-negative detection (1-p_11_), and true-negative detection (1-p_10_) are the complements of ψ, θ_11_, θ_10_, p_11_, and p_10_, respectively.

|  | Intercept | Cover | Depth | X | Y | 2022 | 2023 | Cover:Depth | X:Y |
| --- | --- | --- | --- | --- | --- | --- | --- | --- | --- |
| *ψ*  *(CI)* | 0.45  (0.2, 0.7) | **0.95**  **(0.3, 1.7)** | -0.29  (-0.9, 0.3) | -0.75  (-1.6, 0.1) | **0.94**  **(0.1, 1.7)** | 0.05  (-0.8, 0.9) | -0.32  (-1.2, 0.6) | 0.33  (-0.4, 0.6) | 0.24  (-0.6, 1.0) |
| *PIP* | 1 | **1** | **0.6** | **0.8** | **0.9** | **0.6** | **0.6** | **0.6** | **0.6** |
| θ*_11_*  *(CI)* | 0.89  (0.7, 1.0) | 0.22  (-0.6, 1.0) | -0.08  (-1.0, 1.1) | 0.26  (-2.1, 2.6) | -0.14  (-2.4, 2.1) | -0.33  (-1.8, 1.3) | -0.81  (-2.8, 1.4) | -0.02  (-0.7, 0.8) | 0.37  (-2.1, 2.8) |
| *PIP* | 1 | 0.1 | 0.1 | 0.2 | 0.1 | 0.1 | 0.1 | 0.1 | 0.2 |
| θ*_10_*  *(CI)* | 0.03  (0, 0.1) | 0.69  (-1.9, 3.2) | 0.70  (-1.9, 3.3) | -0.25  (-2.4, 2.0) | 0.38  (-1.8, 2.5) | -0.80  (-3.2, 1.5) | -0.93  (-3.2, 1.3) | -0.29  (-3.0, 2.4) | 1.04  (-0.9, 3.3) |
| *PIP* | 1 | 0.3 | 0.3 | 0.3 | 0.3 | 0.3 | 0.3 | 0.3 | 0.3 |
| *p_11_*  *(CI)* | 0.94  (0.8, 1.0) | **1.07**  **(0.2, 2.1)** | **-1.45**  **(-2.3, -0.8)** | -0.41  (-2.9, 2.2) | 0.72  (-1.8, 3.2) | -0.28  (-2.2, 2.0) | -1.18  (-3.3, 1.3) | 0.01  (-0.9, 0.9) | 0.29  (-2.4, 2.9) |
| *PIP* | 1 | **0.8** | **1** | 0.5 | 0.5 | 0.5 | 0.5 | 0.3 | 0.5 |
| *p_10_*  *(CI)* | 0.02  (0, 0.1) | -0.26  (-1.6, 0.8) | -0.48  (-2.1, 0.7) | -1.35  (-3.2, 0.7) | 1.58  (-0.3, 3.2) | -0.34  (-2.5, 1.5) | -0.30  (-2.2, 1.4) | 0.08  (-1.3, 1.4) | 0.54  (-1.2, 2.5) |
| *PIP* | 1 | 0.2 | 0.2 | 0.5 | **0.6** | 0.2 | 0.2 | 0.1 | 0.2 |
