# Supplementary material for: Navigating uncertainty in environmental DNA detection of a nuisance marine macroalga
Source: PLoS One. 2025 Feb 4;20(2):e0318414. doi: 10.1371/journal.pone.0318414 (PMC11793909; doi:10.1371/journal.pone.0318414)
Supplement: S6 Table — Readiness checklist and validation steps from Thalinger et al. [25] for the Chondria tumulosa quantitative polymerase chain reaction (qPCR) assay using environmental DNA. (DOCX) [file pone.0318414.s006.docx]

**S6 Table. Assay readiness checklist.** Readiness checklist and validation steps from Thalinger et al. [25] for the *Chondria tumulosa* quantitative polymerase chain reaction (qPCR) assay using environmental DNA.

| **Variable to report** | **Definition** | **Level on scale** | **Completed / reported (yes=1 or no=0)** | **This study** |
| --- | --- | --- | --- | --- |
| species name | scientific name | informative | 1 | *Chondria tumulosa* |
| common name | if applicable, name the species is commonly referred to | informative | n/a | n/a |
| broad taxonomic group | higher taxonomic level the target species is associated with | informative | 1 | Rhodomelaceae, Rhodophyta |
| eDNA source | sample type from which eDNA is processed | informative | 1 | Surface seawater |
| assay type | PCR platform | informative | 1 | qPCR |
| gene/region | the genomic region the assay is designed to amplify | informative | 1 | rbcL |
| primer F (5'-3') | forward primer sequence in the 5' to 3' direction | informative | 1 | Table 1 |
| primer R (5'-3') | reverse primer sequence in the 5' to 3' direction | informative | 1 | Table 1 |
| probe (if applicable; 5'-3') | if applicable, probe sequence in the 5' to 3' direction | informative | n/a | n/a |
| 5' reporter dye (e.g. FAM, VIC, TET, NED) | if applicable, name of the used reporter dye | informative | n/a | n/a |
| internal quencher (in the middle of the probe sequence; e.g. ZEN) | if applicable, name of the internal quencher | informative | n/a | n/a |
| 3' quencher (ZEN, BHQ1, BHQ2, IBFQ, TAMARA) | if applicable, name of the 3' quencher | informative | n/a | n/a |
| TaqMan MGBNFQ (minor groove binder non fluorescent quencher) | if applicable, confirm the use of a TaqMan MGBNFQ (1 = yes, 0 = no) | informative | n/a | n/a |
| PCR chemistry | for qPCR assays only: PCR kit(s) used together with the assay | informative | 1 | S3 Table |
| new assay | the assay or parts of it have not been published previously (1 = yes, 0 = no) | informative | 1 | Novel assay |
| modification of previously published assay | the assay or parts of it have been previously published and are now developed further (1 = yes, 0 = no) | informative | n/a | n/a |
| previous publications | please list previous publications the new work is building on | informative | n/a | n/a |
|  |  |  |  |  |
| ***in silico* analysis** | | | |  |
| **minimum criterion: target species** | Primers specifically match target | Level 1 | 1 | See Methods |
| database reported | database primers were compared to (e.g., NCBI) | Level 1 | 1 | NCBI |
| software name | name of software used to conduct *in silico* PCR (e.g., PrimerBlast) | Level 1 | 1 | PrimerBLAST |
| software parameters | parameters set for *in silico* PCR | Level 1 | 1 | See Methods |
| how many mismatches etc. | number of mismatches between primers and closely related species or co-occurring species | Level 1 | 1 | S2 Table |
| where are mismatches located 3' 5' | where mismatches are located | Level 1 | 1 | S2 Table |
| list potential non target organisms that may cause problems | List of non-target species that may cause problems with co-amplification | Level 1 | 1 | S2 Table |
| test closely related species | Primers not likely to amplify closely related species | Level 2 | 1 | See Methods |
| test co-occurring species | Primers not likely to amplify co-occurring species | Level 3 | 1 | See Methods |
| **target tissue testing** | | | |  |
| **minimum criterion: target tissue** | used target tissue for testing assay; does not mean extraction of target tissue needs to be described in the paper. Code as "1" if tested with target tissue extracts. | Level 1 | 1 | See Methods |
| tissue source | collection date information for tissue | Level 1 | cited within | [53] |
| geographic scope | location information for target tissue | Level 1 | 1 | Hawaiʻi |
| number of individuals the tissue was extracted from | number of individuals target tissue is from | Level 1 | cited within | [53] |
| tested on more than one haplotype | tested assay on one or more haplotype(s); If multiple haplotypes are not expected in the geographic range for validation and this is not necessary, then leave blank | Level 1 |  |  |
| DNA concentration of the target extracts | DNA quantification and method of quantification. Attention: DNA concentration of tissue might be reported next to the PCR conditions | Level 1 | 1 | See Methods |
| **PCR (target tissue and eDNA PCR together):** for Level 1, only few very basic PCR reporting criteria are evaluated (marked with L1); for Level 2 and higher, the whole block is taken into account. | | | |  |
| **minimum criterion: primer sequence at level 1; DNA extract volume in PCR at higher levels** | at Level 1: primer sequence included in the manuscript; at Level 2 or higher: the volume of DNA extract going into the PCR must be reported | Level 1 and 2 | 1 | See Methods |
| primer sequences reported; L1 | the primer sequences are contained in the manuscript | Level 1 | 1 | Table 1 |
| thermocycling conditions; L1 | conditions used to amplify target tissue; enough PCR specifications reported to get a basic understanding how the cycler was operated | Level 1 | 1 | S3 Table |
| check of amplification success; L1 | process used to verify amplification success (electrophoresis, melt curve, etc.) | Level 1 | 1 | S1 Figure |
| amplification confirmed by sequencing; L1 | methods used for sequence confirmation and how many positives where correct | Level 1 | 1 | S10 Figure |
| gene / region; L1 | the genomic region the assay is designed to amplify | Level 1 | 1 | rbcL |
| probe (if applicable; 5'-3') | if applicable, probe sequence in the 5' to 3' direction | Level 2 | n/a | n/a |
| 5' reporter dye | if applicable, name of the used reporter dye | Level 2 | n/a | n/a |
| internal quencher | if applicable, name of the internal quencher | Level 2 | n/a | n/a |
| 3' quencher | if applicable, name of the 3' quencher | Level 2 | n/a | n/a |
| taqman MGBNFQ | if applicable, confirm the use of a TaqMan MGBNFQ (1 = yes, 0 = no) | Level 2 | n/a | n/a |
| volume | total volume of each reaction going into PCR | Level 2 | 1 | 10 μL |
| primer concentration | concentration of each primer and probe when applicable | Level 2 | 1 | 10 μM |
| use of commercial Master Mix | if applicable, master mix name and manufacturer | Level 2 | 1 | SSoAdvanced SybrGreen Universal Supermix (Bio-rad) |
| use of custom Master Mix | if applicable, mixed reagents themselves in a specific way | Level 2 | n/a | n/a |
| MgCl2 concentration | concentration reported | Level 2 | n/a | n/a |
| dNTP concentration | concentration reported | Level 2 | n/a | n/a |
| buffer type and concentration | recipe for buffer contents and concentration | Level 2 | n/a | n/a |
| enzyme type and concentration | type and brand of Taq (e.g. Fast Start Taq, Roche, AmpliTaq gold, Applied Biosystems) | Level 2 | n/a | n/a |
| enhancer chemicals (additives TMAC, BSA, DMSO….) | concentration and reason reported | Level 2 | 1 | BSA |
| DNA extract volume in PCR | the volume of DNA extract going into the PCR must be reported | Level 3 | 1 | 1 μL |
| cycler model | make and model of PCR machine | Level 2 | 1 | Bio-Rad CFX96 |
| annealing time | annealing time reported | Level 2 | 1 | S3 Table |
| annealing temperature | annealing temperature reported | Level 2 | 1 | S3 Table |
| cycles | number of cycles reported | Level 2 | 1 | S3 Table |
| technical replication | used PCR technical replication | Level 2 | 1 | 3 |
| optimization of PCR conditions | reporting of any methods used to optimize PCR conditions or protocol | Level 2 | 1 | Touchdown PCR, S3 Table |
| ***in vitro* testing on closely related non-target species** | | | |  |
| **minimum criterion: any *in vitro* non-target tissue testing** | if any of the below criteria is fulfilled, the minimum criterion is fulfilled | Level 2 | 1 | See Methods |
| how many per species | number of tissue samples per non-target species tested | Level 2 |  |  |
| geographic origin | location information for non-target tissue | Level 2 | cited within | [53] |
| tissue source (fresh or old) | collection date information for non-target tissue | Level 2 |  |  |
| non-target amplification checked via sequencing | methods used for sequence confirmation and how many positives where correct | Level 2 | 1 | S10 Figure |
| DNA concentration | method of quantification and amount or range for non-target tissue samples | Level 2 | 1 | See Methods |
| **stray variables:** These are important for the general validation process (total scoring percentage), but do not conform to any of the other blocks. No minimum criterion is associated with them. | | | |  |
| basic geographic scope | Study reports something about the geographic area in which the assay was tested, e.g. ponds in a certain area of the UK | Level 1 | 1 | Shallow reefs of PMNM, HI, USA |
| unidirectional lab flow | Study separated processes by low vs. high quantities of DNA. For example, separate spaces for water filtration, DNA extraction and PCR preparation from post PCR analysis | Level 3 | 1 | See Methods |
| extensive geographic testing | Study reports potential area in which the assay can be used, e.g. the whole species occurrence range | Level 4 | 1 | See Discussion |
| **extraction method performed on environmental DNA samples** | | | |  |
| **minimum criterion: method of extraction** | use of a kit (provide name and manufacturer) or custom (provide all details of chemicals including their manufacturer and concentrations used) | Level 3 | 1 | See Methods |
| modifications from established protocol | if no modifications were included (e.g. from a commercial protocol) , scored as 0. If the protocol was used in a previous publication and changes are reported in detail, scored as 1. | Level 3 | 1 | See Methods |
| lysate volume | volume the filter or pellet is lysed in | Level 3 | 1 | See Methods |
| extraction negative control | use of a negative control for extraction procedure | Level 3 | 0 |  |
| elution volume | volume the DNA is reconstituted in at the end of extraction | Level 3 | 1 | See Methods |
| verification of DNA extraction | a quantification of DNA was performed or electrophoresis was performed | Level 3 | 0 |  |
| **concentration of eDNA from environmental sample** | | | |  |
| **minimum criterion: filter type OR precipitation chemicals** | either the filter type or the precipitation chemicals must be provided | Level 3 | 1 | See Methods |
| volume/mass of environmental sample | for water samples, that the water volume per sample, for soil/ sediment the mass | Level 3 | 1 | 2 L |
| filter type | if applicable, the brand and material type of filter | Level 3 | 1 | Mixed cellulose ester (Millipore) |
| pore size | size of pores | Level 3 | 1 | 0.22 micron |
| surface area / diameter | size of filter | Level 3 | 1 | 47 mm |
| pressure used for filtration | any estimate for the pressure used to pump the water sample past the filter membrane | Level 3 | 0 |  |
| filter preservation | filter preservative used to store filter before extraction; freezing alone does not count | Level 3 | 0 | Freezing in liquid nitrogen |
| precipitation chemicals added | if applicable, concentrations of chemicals used to precipitate DNA | Level 3 | n/a | n/a |
| centrifugation force and time for precipitation | time and force used to precipitate DNA | Level 3 | n/a | n/a |
| temperature of precipitation | temperature the centrifuge was kept at during centrifugation | Level 3 | n/a | n/a |
| storage between collection and processing | filters placed at a specific storage temperature or location before proceeding to extraction | Level 3 | 1 | Stored on ice |
| "field" blanks | use of negative controls for filtration process | Level 3 | 1 | 1 per site |
| **detection obtained from environmental samples** | | | |  |
| **minimum criterion: detection from a water sample (artificial or natural habitat)** | either natural habitat, artificial habitat, or DNA spiked into sample must be positive | Level 3 | 1 | Natural habitat |
| criteria to determine detection | for example, a certain number of positive amplifications / bands per sample | Level 3 | 1 | See Methods |
| sample type (e.g. water sediment) | type of environmental sample the DNA was extracted from | Level 3 | 1 | Surface seawater |
| artificial habitat | samples taken from a mesocosm tank in lab or enclosure in zoo etc., including semi-natural systems | Level 3 | n/a | n/a |
| natural habitat | samples taken from locations uncontrolled by researchers | Level 3 | 1 | Shallow coral reefs |
| target DNA spiked into sample type | water sample where the species DNA was added such as tap water or water from a natural habitat | Level 3 | n/a | n/a |
| environmental variables recorded | any record of general conditions at the sampling location helping to understand eDNA detection (e.g. pH, conductivity, microbial activity, etc.) | Level 3 | 1 | S1 Table |
| sequence validation of positive environmental samples | reporting sequencing of field detections at least for some percentage of the positive amplicons; also applies to positive field blanks | Level 3 | 1 | S10 Figure |
| **limit of detection established (LOD)** | | | |  |
| **minimum criterion: determined limit of detection** | was a limit of detection (LOD) established? Information might not be termed "LOD" in the papers | Level 4 | 1 | 160 copies |
| method of establishment | may not be called LOD, but authors define a DNA concentration using some method below which the amplification is not different from zero | Level 4 |  | See Methods |
| multiple replicates of standard curve | was the LOD value determined through repetition (e.g., the standard curve run more than once) | Level 4 |  | Seven triplicate 10-fold dilutions per qPCR sample plate |
| **extensive field testing of environmental samples** | | | |  |
| **minimum criterion: multiple locations or multiple samples** | either multiple locations or multiple samples needs to be positive | Level 4 | 1 | See Results |
| multiple locations | use of locations from more than a single environmental entity (e.g water body, a single river, a single meadow) | Level 4 | 1 | Figure 1 |
| site where species is not there | site where species is not present | Level 4 | 1 | Figure 1 |
| site where species is there | site where species is present | Level 4 | 1 | Figure 1 |
| site where species was there historically | if applicable, site where the species has been lost from | Level 4 | 1 | Figure 1 |
| multiple samples at each site | biological replicate samples from the same environmental entity | Level 4 | 1 | 2-3 replicates, S1 Table |
| environmental gradients (e.g. abiotic/biotic conditions) | sites within or among environmental entities | Level 4 | 1 | S1 Table |
| different known target species densities | species varied in density between sample locations | Level 4 | 1 | S1 Table |
| testing against data from traditional methods | compared to a conventional method of detection; the conventional method results don't need to be from the same day; it's enough if there is some record | Level 4 | 1 | S1 Table |
| inhibition testing | determined environmental variables that can cause a change in amplification | Level 4 | 1 | See Results |
| ***in vitro* testing on co-occurring non-target species** | | | |  |
| **minimum criterion: any advanced *in vitro* testing undertaken** | if any of the below criteria is fulfilled, the minimum criterion is fulfilled | Level 4 |  |  |
| geographic region tested | what geographic region was considered for defining co-occurring (e.g. a few sites, or large range such as known species range) | Level 4 | 1 | Known species range and additional suspected sites |
| source of species occurrence information | information from grey literature, standardized surveys, gbif database, etc. | Level 4 | 1 | Standardized visual surveys |
| multiple samples per species | number of individuals from each non-target species | Level 4 | 1 | [53] |
| geographic origin of tissue samples | location information for target tissue; more than one sample from one site tested | Level 4 | 1 | [53] |
| tissue source | collection date information for non-target tissue | Level 4 | 1 | [53] |
| non-target amplification checked via sequencing | methods used for sequence confirmation and how many positives where correct | Level 4 | 1 | S10 Figure |
| DNA concentration | method of quantification and amount or range for non-target tissue samples | Level 4 | 1 | See Methods |
| **comprehensive specificity testing** | | | |  |
| **minimum criterion: non co-occurring/closely related species checked from *in silico*** | any tests of unexpected taxa that match *in silico*, but not co-occurring or are from a taxonomic group highly divergent from species (e.g., bacteria, fungi, etc.) | Level 5 | 1 | S2 Table |
| justify non-verified results from *in silico* (geographic/realistic) | provide reasons for not checking an *in silico* match (e.g., narrow species distribution on another continent) | Level 5 | 1 | Closest match outside of Pacific basin |
| non-target amplification checked via sequencing | methods used for sequence confirmation and how many positives where correct | Level 5 | 1 | See Methods, S10 Figure |
| **detection probability estimation from statistical modelling** | | | |  |
| **minimum criterion: any effort made towards detection probability estimation** | if any of the below criteria is fulfilled, the minimum criterion is fulfilled | Level 5 |  |  |
| sampling through time | samples taken at same location in time | Level 5 |  |  |
| sampling spatially | sample taken throughout an expected range of the species presence, but does not have to be an entire species range | Level 5 | 1 |  |
| statistical modelling | applied modeling of some sort to estimate detection probabilities in space or time | Level 5 | 1 | Site-occupancy detection modeling |
| occupancy modelling | applied modelling to estimate occupancy | Level 5 | 1 | Site-occupancy detection modeling |
| parameters and variables of model reported | all assumptions and parameters/variables stated | Level 5 | 1 | See Methods |
| **understanding ecological and physical factors influencing eDNA in the environment** | | | |  |
| **minimum criterion: single factor influencing eDNA in the environment tested** | if any of the below criteria is fulfilled, the minimum criterion is fulfilled | Level 5 |  |  |
| known origin (e.g. shedding rate) | direct estimates from species during life stages or other transitions (eating, resting, etc.) | Level 5 |  |  |
| state | determined the condition of DNA from which detection arises (e.g., particle bound, cell or organelle, free DNA) | Level 5 |  |  |
| degradation rate | determined through experiments of species presence and removal from a system | Level 5 |  |  |
| transport | movement of DNA originating from a species in a natural water body | Level 5 |  |  |
