# Supplementary material for: Navigating uncertainty in environmental DNA detection of a nuisance marine macroalga
Source: PLoS One. 2025 Feb 4;20(2):e0318414. doi: 10.1371/journal.pone.0318414 (PMC11793909; doi:10.1371/journal.pone.0318414)
Supplement: S1 Fig — Assay development consisted of a temperature gradient approach on standard samples of DNA extracted from Chondria tumulosa tissue and no-template controls (NTC) to monitor for contamination. (a) The ideal annealing temperature (54.7–64°C) was selected among triplicate amplifications that produced a sigmoidal curve in the fewest cycles. (b) Melt curves were verified to only produce a single tall, narrow peak. (c) Threshold cycle (Cq) values are plotted against triplicate DNA serial dilution starting concentrations to generate a best fit standard curve (R2 = 0.999, slope = -3.79, intercept = 33.62), using the curve-fitting approach [63]. Synthetic C. tumulosa DNA was quantified using a fluorometer (Qubit, Invitrogen) and diluted to create 10-fold serial dilutions ranging from 106 to 100 copies per reaction. The limit of detection (LOD, where 95% of technical replicates amplify) is marked with a solid red line and the limit of quantification (LOQ, the lowest initial DNA concentration quantifiable with a coefficient of variation below 35%) is marked with a dashed line. (DOCX) [file pone.0318414.s007.docx]

**
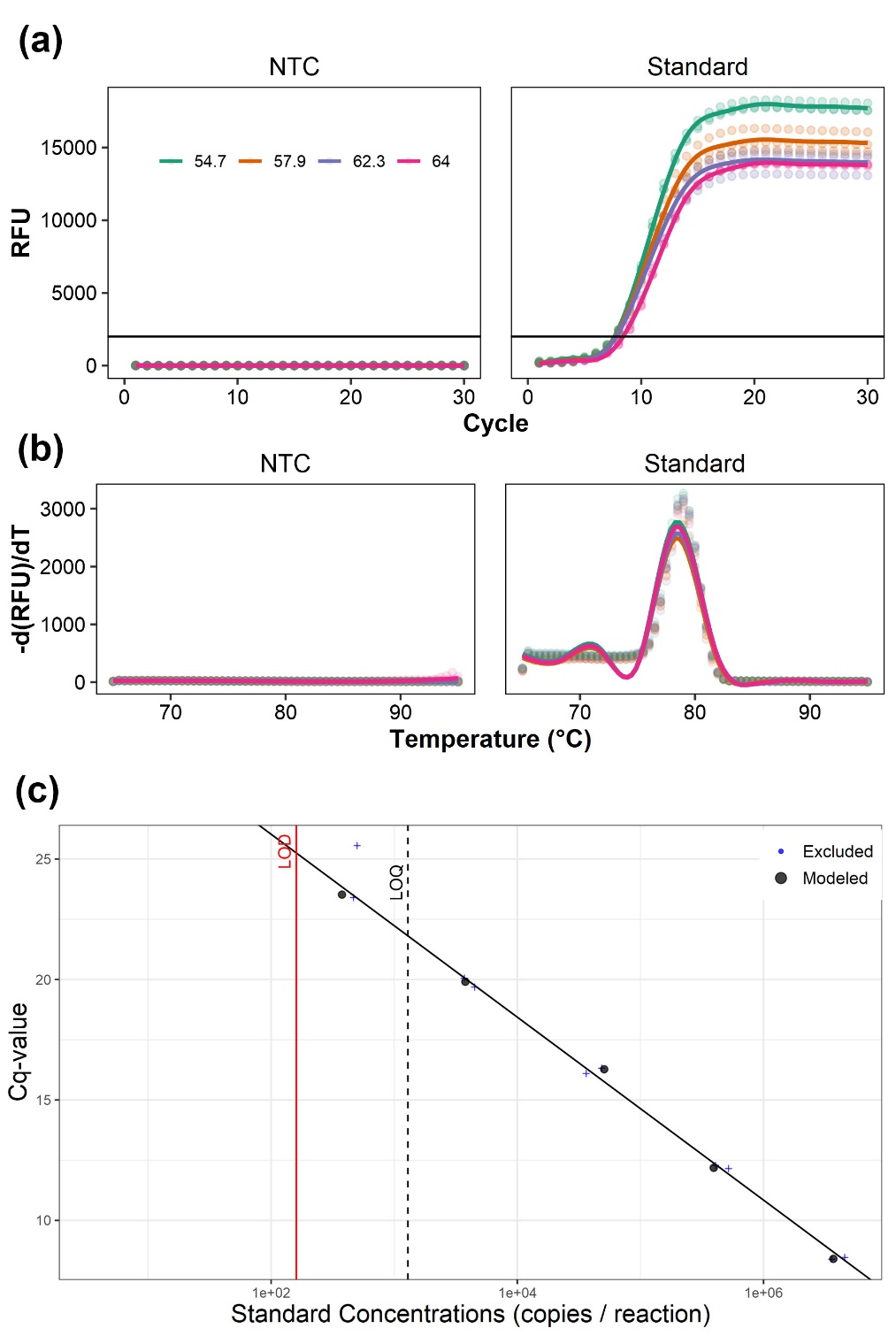
**

**S1 Figure. Assay validation with temperature gradient and standard curve.** Assay development consisted of a temperature gradient approach on standard samples of DNA extracted from *Chondria tumulosa* tissue and no-template controls (NTC) to monitor for contamination. (a) The ideal annealing temperature (54.7-64 °C) was selected among triplicate amplifications that produced a sigmoidal curve in the fewest cycles. (b) Melt curves were verified to only produce a single tall, narrow peak. (c) Threshold cycle (C_q_) values are plotted against triplicate DNA serial dilution starting concentrations to generate a best fit standard curve (R^2^=0.999, slope=-3.79, intercept=33.62), using the curve-fitting approach [63]. Synthetic *C. tumulosa* DNA was quantified using a fluorometer (Qubit, Invitrogen) and diluted to create 10-fold serial dilutions ranging from 10^6^ to 10^0^ copies per reaction. The limit of detection (LOD, where 95% of technical replicates amplify) is marked with a solid red line and the limit of quantification (LOQ, the lowest initial DNA concentration quantifiable with a coefficient of variation below 35%) is marked with a dashed line.
