# Supplementary material for: Navigating uncertainty in environmental DNA detection of a nuisance marine macroalga
Source: PLoS One. 2025 Feb 4;20(2):e0318414. doi: 10.1371/journal.pone.0318414 (PMC11793909; doi:10.1371/journal.pone.0318414)
Supplement: S2 Fig — Adapted from Griffin et al. [38] of the hierarchical site-occupancy model implemented in the RShiny application [66]. Environmental DNA is collected from S independent sites using M independent water samples, analyzed using K independent quantitative polymerase chain reactions (qPCR) replicates. Incidental observations confirming target species presence is denoted as k with a common probability of confirmed detection, π. For every sth site and mth water sample, a positive qPCR result is denoted as ysm. The occupancy state defines eDNA presence-absence at a site, z, or in a sample, w. The probability of eDNA being present at a site, ѱ, captured in a sample, θ, or detected in an individual qPCR replicate, p, are estimated. More specifically, the model estimates field (stage 1) capture using the probability of eDNA presence in a sample from a site is θ11 if the site was occupied and θ10 if it was unoccupied (with false-negatives and true negatives being 1-θ11 and 1-θ10, respectively). Laboratory (stage 2) detection is estimated with positive qPCR replicates from samples containing target eDNA, p11, or not containing target eDNA, p10 (with false negatives and true negatives being 1-p11 and 1-p10, respectively). (DOCX) [file pone.0318414.s008.docx]

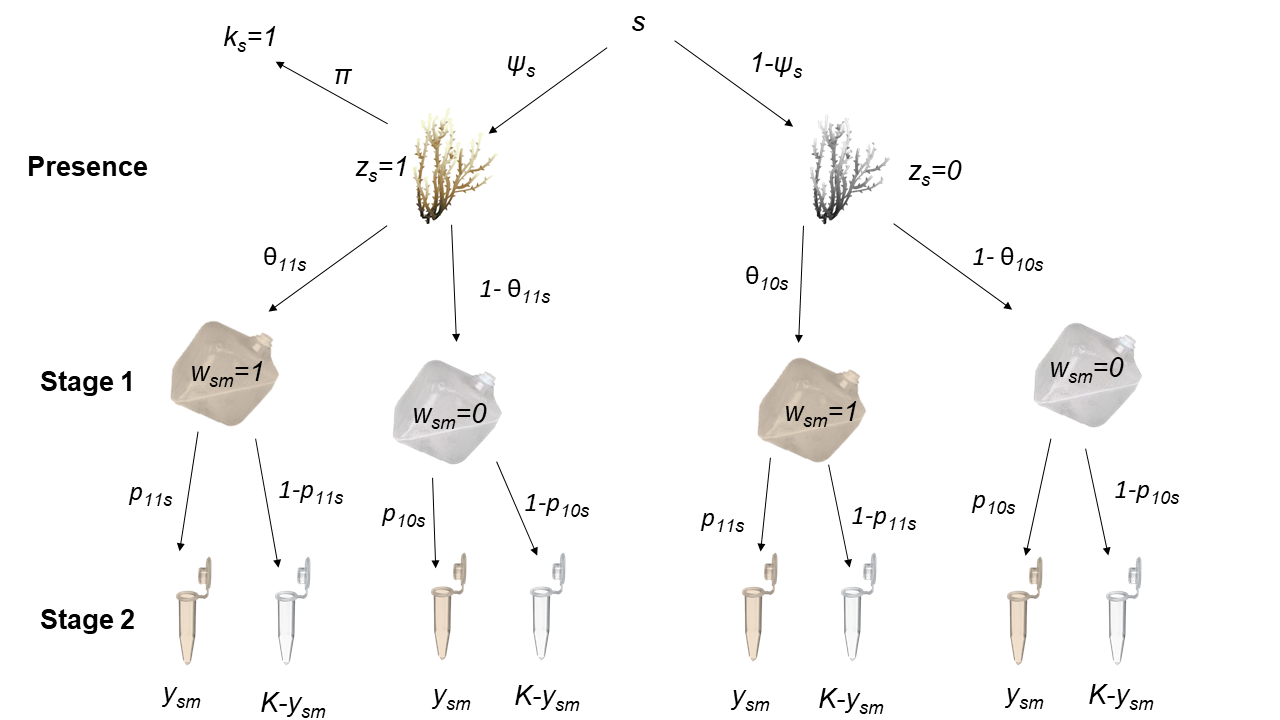


**S2 Figure. Schematic representation of eDNA site-occupancy model framework.** adapted from Griffin et al. [38] of the hierarchical site-occupancy model implemented in the RShiny application [66]. Environmental DNA is collected from *S* independent sites using *M* independent water samples, analyzed using *K* independent quantitative polymerase chain reactions (qPCR) replicates. Incidental observations confirming target species presence is denoted as *k* with a common probability of confirmed detection, *π*. For every *s*th site and *m*th water sample, a positive qPCR result is denoted as *y_sm_*. The occupancy state defines eDNA presence-absence at a site, *z*, or in a sample, *w*. The probability of eDNA being present at a site, *ѱ*, captured in a sample, *θ*, or detected in an individual qPCR replicate, *p*, are estimated. More specifically, the model estimates field (stage 1) capture using the probability of eDNA presence in a sample from a site is *θ_11_* if the site was occupied and *θ_10_* if it was unoccupied (with false-negatives and true negatives being *1-θ_11_* and *1-θ_10_*, respectively). Laboratory (stage 2) detection is estimated with positive qPCR replicates from samples containing target eDNA, *p_11_*, or not containing target eDNA, *p_10_* (with false negatives and true negatives being *1-p_11_* and *1-p_10_*, respectively).
