# Supplementary material for: Navigating uncertainty in environmental DNA detection of a nuisance marine macroalga
Source: PLoS One. 2025 Feb 4;20(2):e0318414. doi: 10.1371/journal.pone.0318414 (PMC11793909; doi:10.1371/journal.pone.0318414)
Supplement: S4 Fig — Posterior summaries of the probability of baseline environmental DNA (eDNA) sample capture given presence of target eDNA at a site (θ11) resulting from site-occupancy detection modeling using the RShiny application. (DOCX) [file pone.0318414.s010.docx]

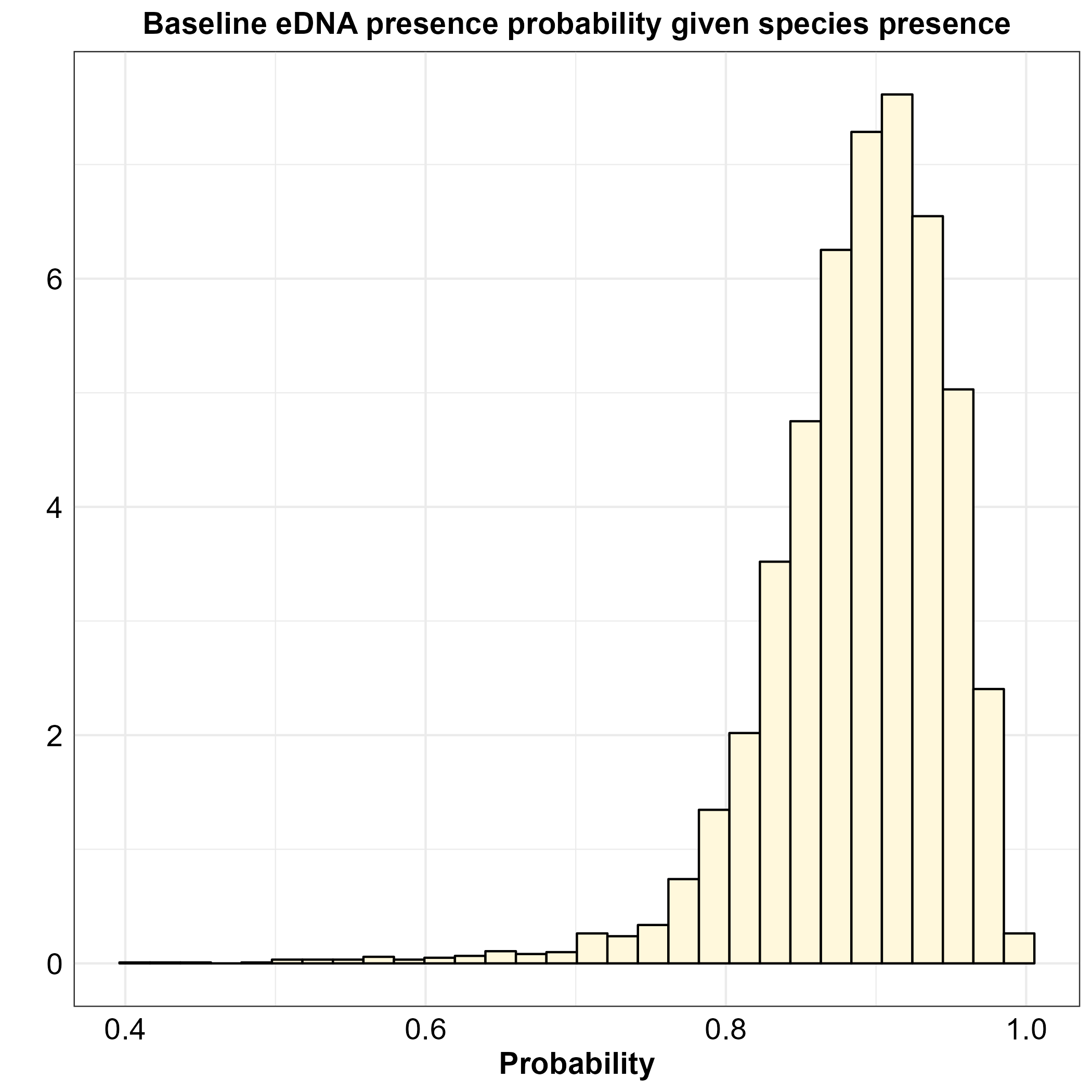


**S4 Figure. Posterior summary of capture.** Posterior summaries of the probability of baseline environmental DNA (eDNA) sample capture given presence of target eDNA at a site (θ_11_) resulting from site-occupancy detection modeling using the RShiny application.
