# Supplementary material for: Navigating uncertainty in environmental DNA detection of a nuisance marine macroalga
Source: PLoS One. 2025 Feb 4;20(2):e0318414. doi: 10.1371/journal.pone.0318414 (PMC11793909; doi:10.1371/journal.pone.0318414)
Supplement: S5 Fig — Posterior summaries of the probability of baseline environmental DNA (eDNA) qPCR replicate detection given presence of target eDNA in a sample (p11) resulting from site-occupancy detection modeling using the RShiny application. (DOCX) [file pone.0318414.s011.docx]

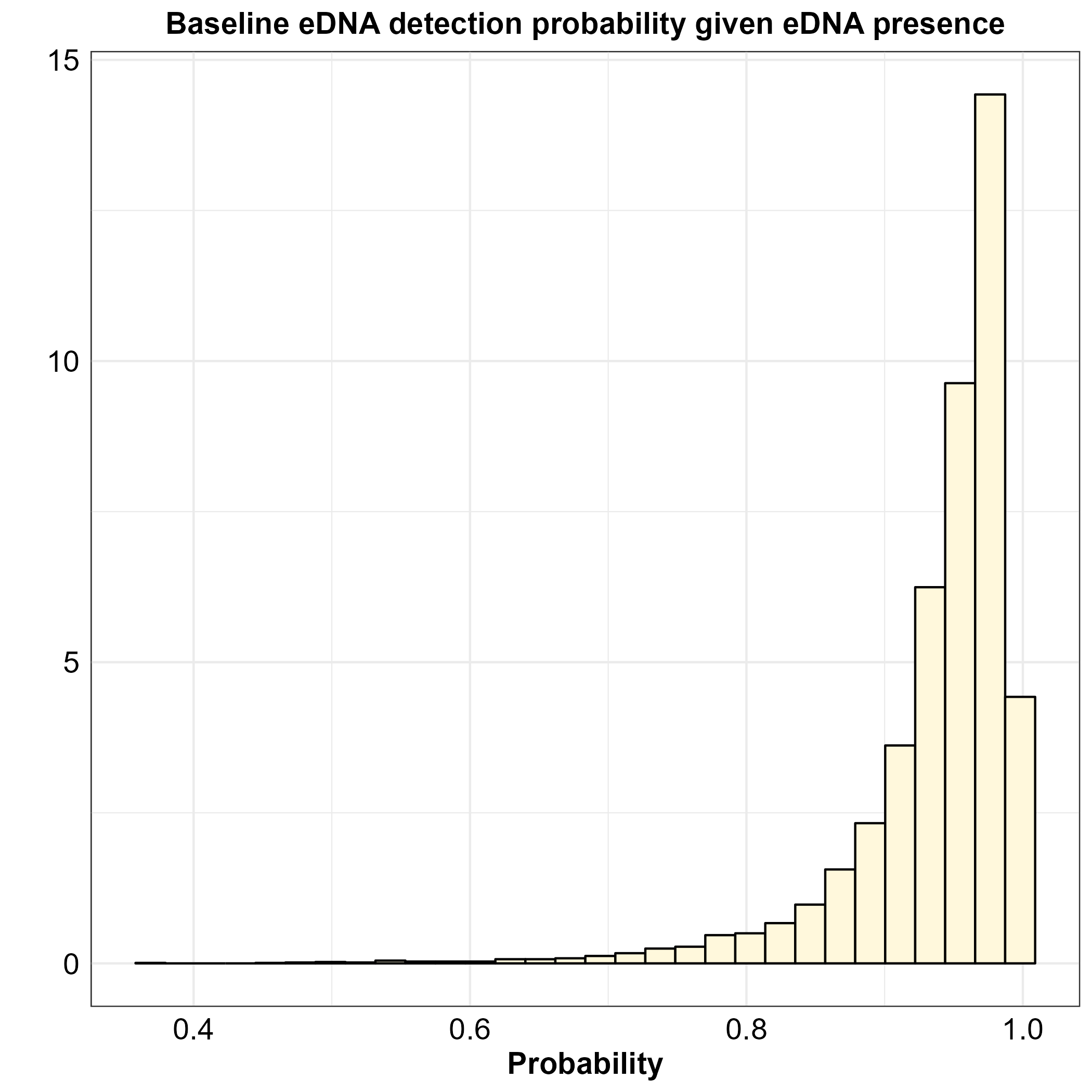


**S5 Figure. Posterior summary of detection.** Posterior summaries of the probability of baseline environmental DNA (eDNA) qPCR replicate detection given presence of target eDNA in a sample (p_11_) resulting from site-occupancy detection modeling using the RShiny application.
