# Supplementary material for: Navigating uncertainty in environmental DNA detection of a nuisance marine macroalga
Source: PLoS One. 2025 Feb 4;20(2):e0318414. doi: 10.1371/journal.pone.0318414 (PMC11793909; doi:10.1371/journal.pone.0318414)
Supplement: S6 Fig — Environmental DNA (eDNA) probability of site occupancy (ψ) model fit trace plot, effective sample sizes (ESS), and Geweke diagnostics from eDNA site-occupancy detection modeling using the RShiny application. (DOCX) [file pone.0318414.s012.docx]

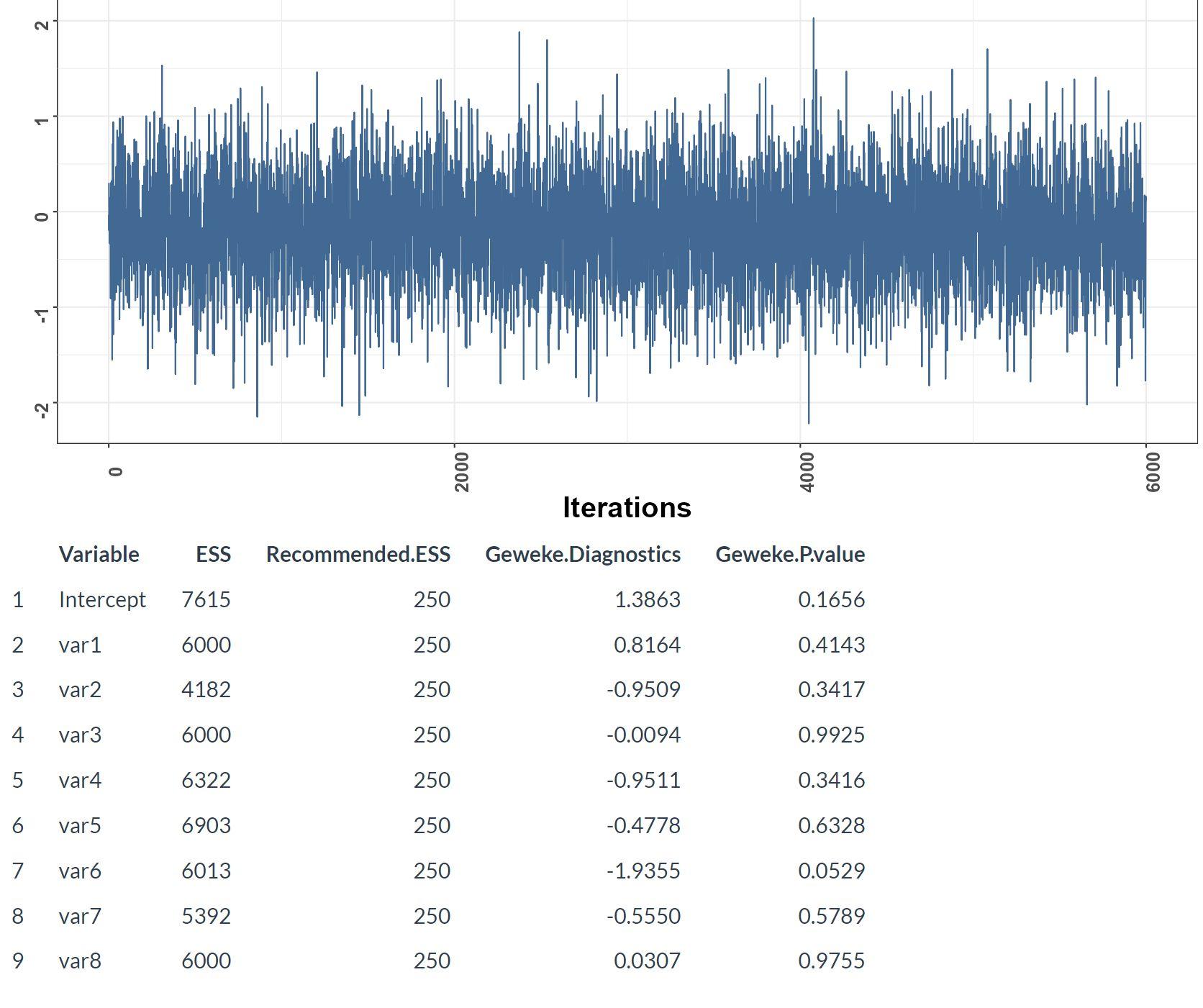


**S6 Figure. Trace plot of occupancy.** Environmental DNA (eDNA) probability of site occupancy (ψ) model fit trace plot, effective sample sizes (ESS), and Geweke diagnostics from eDNA site-occupancy detection modeling using the RShiny application.
