# Supplementary material for: Navigating uncertainty in environmental DNA detection of a nuisance marine macroalga
Source: PLoS One. 2025 Feb 4;20(2):e0318414. doi: 10.1371/journal.pone.0318414 (PMC11793909; doi:10.1371/journal.pone.0318414)
Supplement: S9 Fig — Environmental DNA (eDNA) quantitative polymerase chain reaction (qPCR) amplification curves and visual benthic categorization from across the Hawaiian Archipelago. Sites with corresponding visual data (H01 through L04) were from Hōlanikū (“H”, or Kure Atoll), Kuaihelani (“K”, or Midway Island), Manawai (“M”, or Pearl & Hermes Atoll), Kapou (“KAP”, or Lisianski Island), and Lalo (“L”, or French Frigate Shoals). Sites lacking visual data (OA01 through OA17) were from Oʻahu (“OA”). Amplification (relative fluorescence units, RFU) of Chondria tumulosa eDNA is marked with circles (representing each individual PCR replicate) and a solid line generalized additive model smoother of triplicate PCR reactions among water samples from each site. Line colors refer to the visual categorization of sites from visual surveys (green: “Absent”, orange: “Present”). Control samples, consisting of positive C. tumulosa tissue extractions (“Standard”), equipment blanks (“EB”), and qPCR no-template controls (“NTC”) are marked in black. The mean fluorescence quantification threshold is marked with a dashed grey line. The standard error of the mean (± SE) is shaded, but is often so narrow that it becomes obscured by the curve. (DOCX) [file pone.0318414.s015.docx]

**
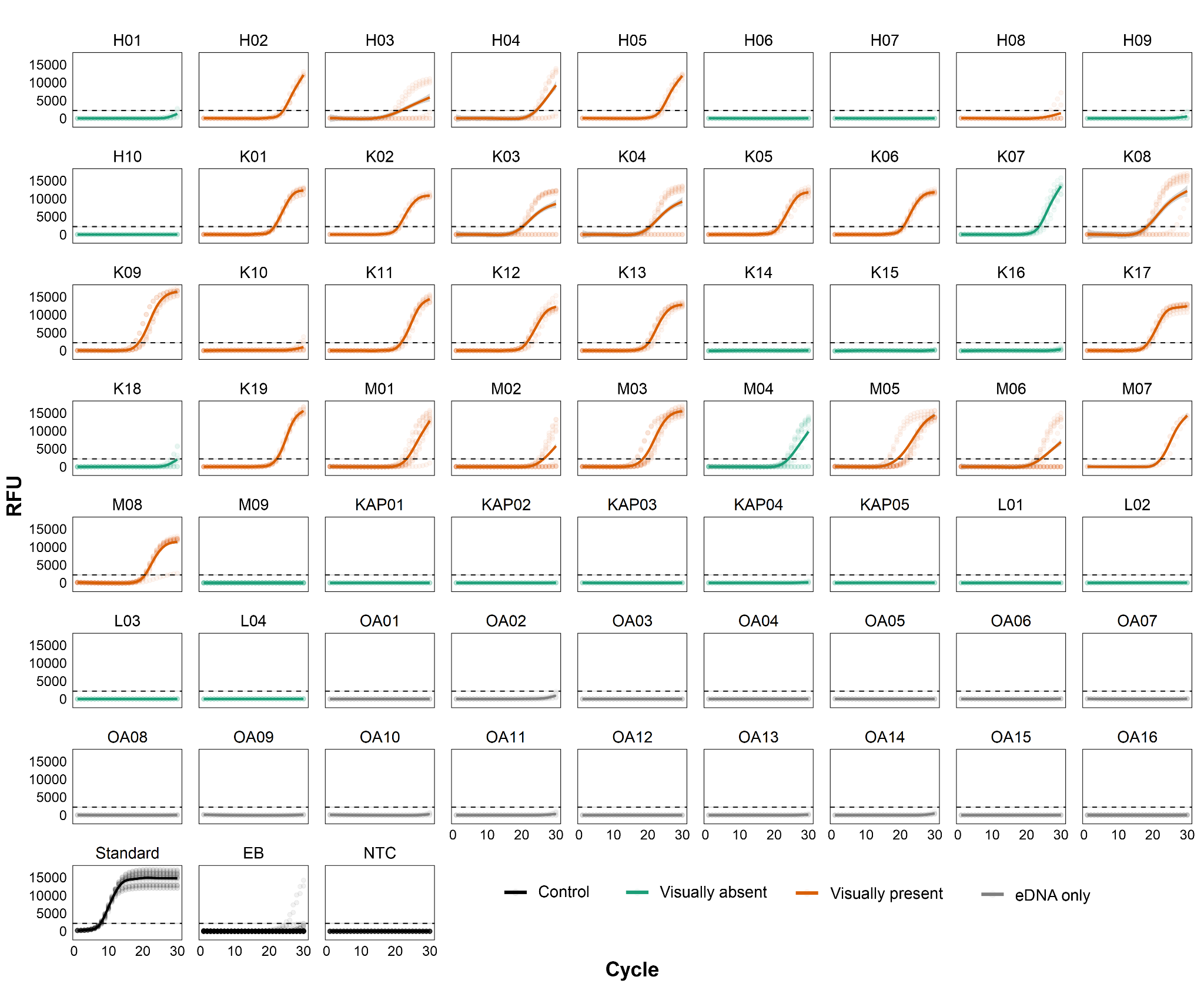
**

**S9 Figure. Amplification curves for surveyed sites across the Hawaiian Archipelago.** Environmental DNA (eDNA) quantitative polymerase chain reaction (qPCR) amplification curves and visual benthic categorization from across the Hawaiian Archipelago. Sites with corresponding visual data (H01 through L04) were from Hōlanikū (“H”, or Kure Atoll), Kuaihelani (“K”, or Midway Island), Manawai (“M”, or Pearl & Hermes Atoll), Kapou (“KAP”, or Lisianski Island), and Lalo (“L”, or French Frigate Shoals). Sites lacking visual data (OA01 through OA17) were from Oʻahu (“OA”). Amplification (relative fluorescence units, RFU) of *Chondria tumulosa* eDNA is marked with circles (representing each individual PCR replicate) and a solid line generalized additive model smoother of triplicate PCR reactions among water samples from each site. Line colors refer to the visual categorization of sites from visual surveys (green: “Absent”, orange: “Present”). Control samples, consisting of positive *C. tumulosa* tissue extractions (“Standard”), equipment blanks (“EB”), and qPCR no-template controls (“NTC”) are marked in black. The mean fluorescence quantification threshold is marked with a dashed grey line. The standard error of the mean (± SE) is shaded, but is often so narrow that it becomes obscured by the curve.
