# Supplementary material for: Navigating uncertainty in environmental DNA detection of a nuisance marine macroalga
Source: PLoS One. 2025 Feb 4;20(2):e0318414. doi: 10.1371/journal.pone.0318414 (PMC11793909; doi:10.1371/journal.pone.0318414)
Supplement: S10 Fig — Environmental DNA index (normalized using the inversed order of operations of the Wisconsin double-standardization) from molecular operational taxonomic units (MOTUs) that were amplified and sequenced using the assay. Amplicons were sequenced on an Illumina MiSeq platform and matched with NCBI’s BLASTn algorithm (%ID > 97, E-value < 1e-10). Chondria tumulosa matched 17 MOTUs and six MOTUs matched to the family Rhodomelaceae, tribe Polysiphonieae. Bar colors refer to the categorization of C. tumulosa from visually surveyed sites (green: “Absent”, orange: “Present”). Samples were from Kuaihelani (“K”, or Midway Island), Manawai (“M”, or Pearl & Hermes Atoll), or Oʻahu (“OA”). (DOCX) [file pone.0318414.s016.docx]

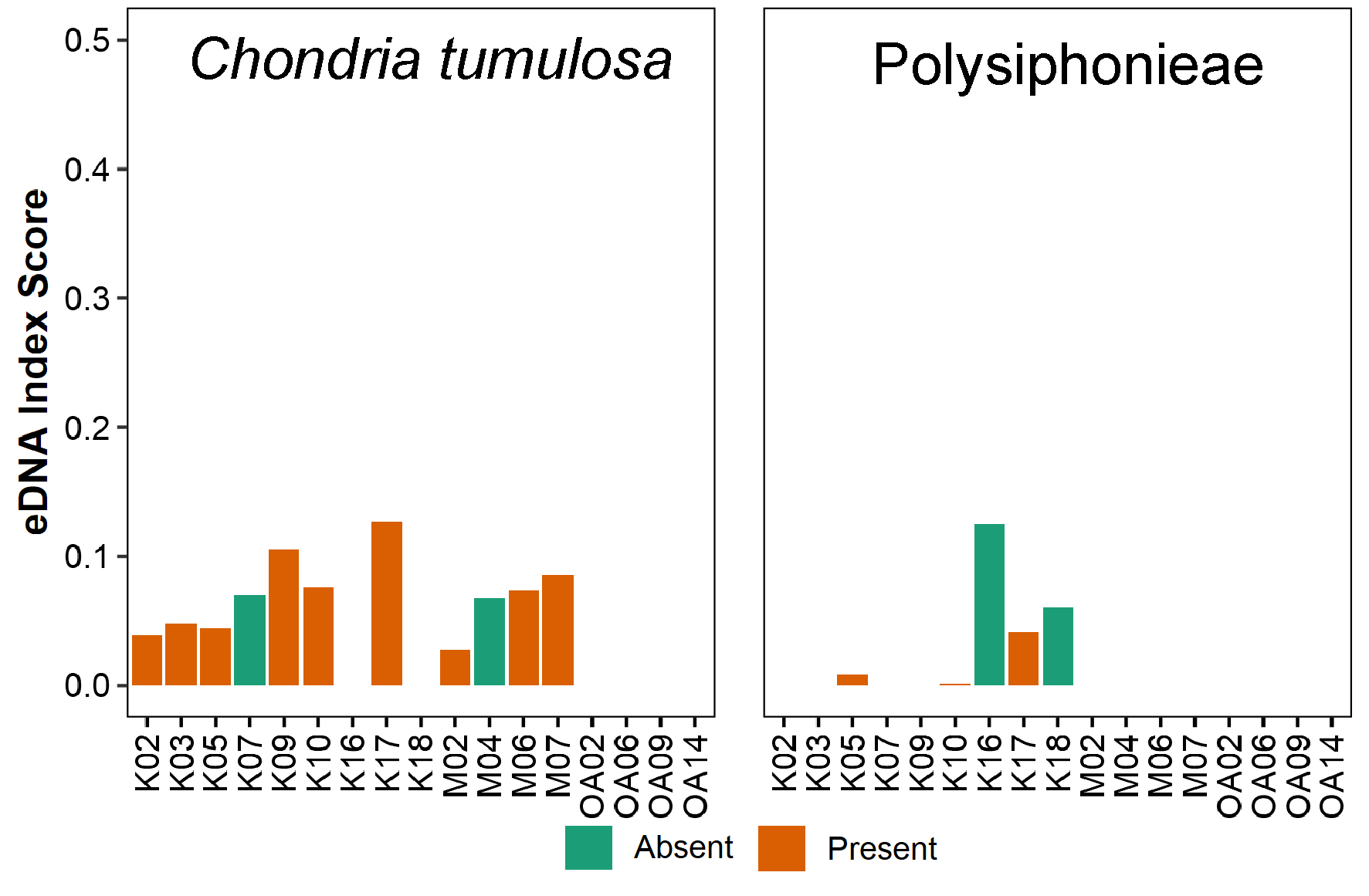


**S10 Figure. Normalized eDNA reads from high-throughput sequencing of samples.** Environmental DNA index (normalized using the inversed order of operations of the Wisconsin double-standardization) from molecular operational taxonomic units (MOTUs) that were amplified and sequenced using the assay. Amplicons were sequenced on an Illumina MiSeq platform and matched with NCBI’s BLASTn algorithm (%ID > 97, E-value < 1e-10). *Chondria tumulosa* matched 17 MOTUs and six MOTUs matched to the family Rhodomelaceae, tribe Polysiphonieae. Bar colors refer to the categorization of *C. tumulosa* from visually surveyed sites (green: “Absent”, orange: “Present”). Samples were from Kuaihelani (“K”, or Midway Island), Manawai (“M”, or Pearl & Hermes Atoll), or Oʻahu (“OA”).
