# Supplementary material for: Navigating uncertainty in environmental DNA detection of a nuisance marine macroalga
Source: PLoS One. 2025 Feb 4;20(2):e0318414. doi: 10.1371/journal.pone.0318414 (PMC11793909; doi:10.1371/journal.pone.0318414)
Supplement: S11 Fig — Example validation of positive detections using quantitative polymerase chain reaction (qPCR) (a) amplification (relative fluorescence units, RFU) curves and (b) melt curve analysis. Panels depict DNA extracted from positive control Chondria tumulosa tissue (Standard, n = 39), no-template controls (NTC, n = 39), equipment blanks (“EB”, n = 63), an exemplar field positive detection at Kuaihelani (K17), and an ambiguous field site which amplified DNA from an unknown alga in the tribe Polysiphonieae (K18). The mean (± standard error of the mean) fluorescence quantification threshold is marked with a dashed black line. Low-level contamination was detected in EB samples from two sites at Kuaihelani (K09 & K14); each had a single technical qPCR replicate amplify above the fluorescence threshold. (DOCX) [file pone.0318414.s017.docx]

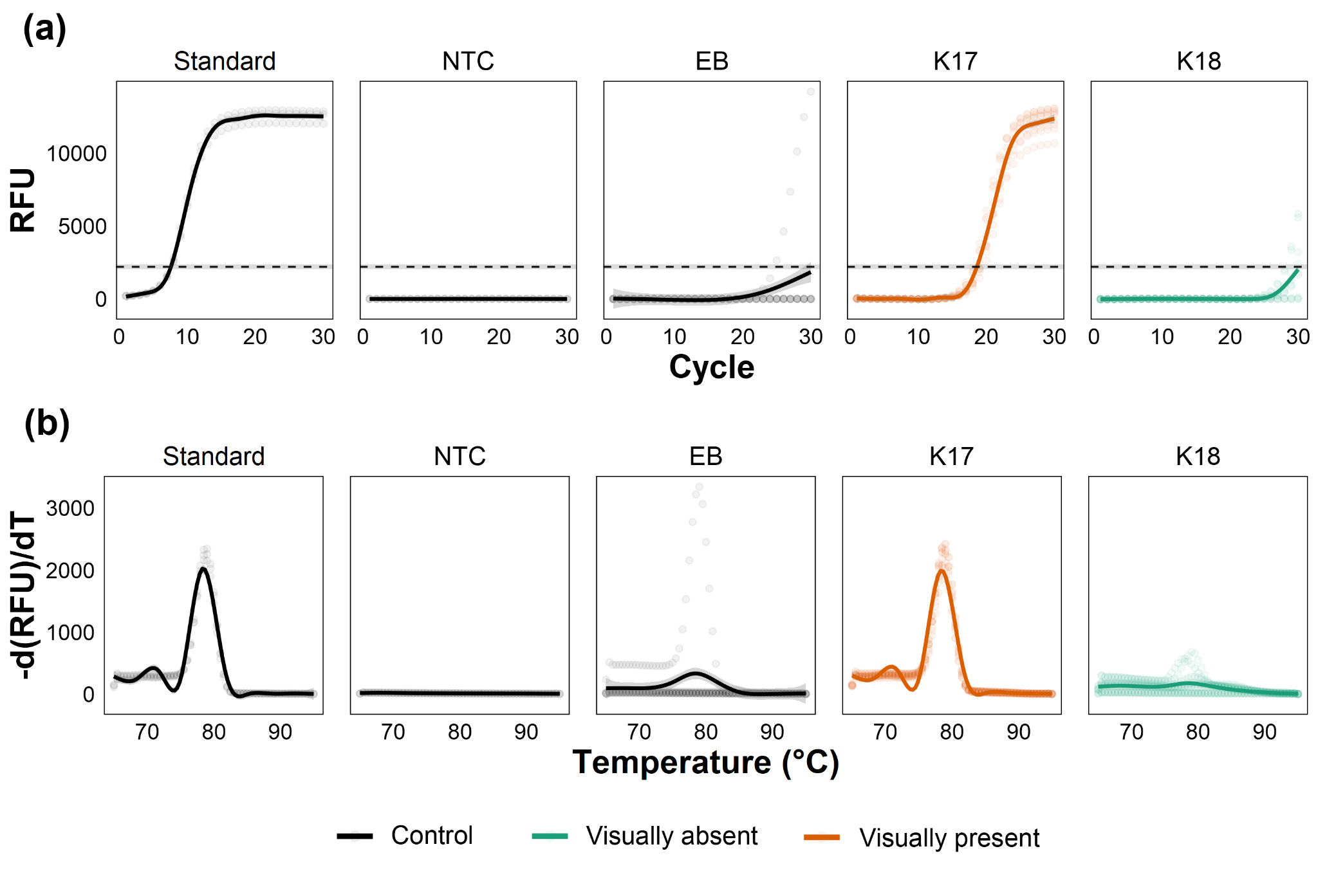


**S11 Figure. Validation of qPCR amplification and melt curves.** Example validation of positive detections using quantitative polymerase chain reaction (qPCR) (a) amplification (relative fluorescence units, RFU) curves and (b) melt curve analysis. Panels depict DNA extracted from positive control *Chondria tumulosa* tissue (Standard, n=39), no-template controls (NTC, n=39), equipment blanks (“EB”, n=63), an exemplar field positive detection at Kuaihelani (K17), and an ambiguous field site which amplified DNA from an unknown alga in the tribe Polysiphonieae (K18). The mean (± standard error of the mean) fluorescence quantification threshold is marked with a dashed black line. Low-level contamination was detected in EB samples from two sites at Kuaihelani (K09 & K14); each had a single technical qPCR replicate amplify above the fluorescence threshold.
